# When is lethal deceptive pollination maintained? A population dynamics approach

**DOI:** 10.1093/aob/mcae108

**Published:** 2024-08-02

**Authors:** Takefumi Nakazawa, Tetsuya K Matsumoto, Koki R Katsuhara

**Affiliations:** Department of Life Sciences, National Cheng Kung University, Tainan 701, Taiwan; Faculty of Environmental, Life, Nature Science and Technology, Okayama University, Okayama 700-8530, Japan; Graduate School of Science and Engineering, Ibaraki University, Mito 310-8512, Japan; Faculty of Environmental, Life, Nature Science and Technology, Okayama University, Okayama 700-8530, Japan

**Keywords:** Alternative stable states, deer herbivory, floral mimicry, forest disturbance, habitat fragmentation, jack-in-the-pulpit, myophily, pitfall-trap flower, population dynamics model, sexual mimicry, rare plant poaching, threatened species

## Abstract

**Background and Aims:**

Not all plant–pollinator interactions are mutualistic, and in fact deceptive pollination systems are widespread in nature. The genus *Arisaema* has a pollination system known as lethal deceptive pollination, in which plants not only attract pollinating insects without providing any rewards, but also trap them until they die. Many *Arisaema* species are endangered from various disturbances, including reduction in forest habitat, modification of the forest understorey owing to increasing deer abundance, and plant theft for horticultural cultivation. We aimed to theoretically investigate how lethal deceptive pollination can be maintained from a demographic perspective and how plant and pollinator populations respond to different types of disturbance.

**Methods:**

We developed and analysed a mathematical model to describe the population dynamics of a deceptive plant species and its victim pollinator. Calibrating the model based on empirical data, we assessed the conditions under which plants and pollinators could coexist, while manipulating relevant key parameters.

**Key Results:**

The model exhibited qualitatively distinct behaviours depending on certain parameters. The plant becomes extinct when it has a low capability for vegetative reproduction and slow transition from male to female, and plant–insect co-extinction occurs especially when the plant is highly attractive to male insects. Increasing deer abundance has both positive and negative effects because of removal of other competitive plants and diminishing pollinators, respectively. Theft for horticultural cultivation can readily threaten plants whether male or female plants are frequently collected. The impact of forest habitat reduction may be limited compared with that of other disturbance types.

**Conclusions:**

Our results have emphasized that the demographic vulnerability of lethal deceptive pollination systems would differ qualitatively from that of general mutualistic pollination systems. It is therefore important to consider the demographics of both victim pollinators and deceptive plants to estimate how endangered *Arisaema* populations respond to various disturbances.

## INTRODUCTION

Pollination plays a vital role in maintaining biodiversity and ecosystem service (Bascompte and Jordano, 2014; Bronstein, 2015). Currently, plant–pollinator relationships face threats from various human-induced disturbances (Kearns *et al.*, 1998; Vanbergen and the Insect Pollinators Initiative, 2013; Neuschulz *et al.*, 2016). Nevertheless, our understanding of plant–pollinator population dynamics and their responses to disturbance remains limited. This is primarily because the mechanisms underlying the stability of plant–pollinator relationships have traditionally been studied mainly on an evolutionary time scale (Pellmyr and Huth, 1994; Frederickson, 2013). Meanwhile, population dynamics models can help understand how the systems respond to environmental changes on the time scale of population dynamics, thereby providing useful insights into conservation management (Lande *et al.*, 2003; Nakazawa, 2014). Therefore, some theoretical studies have recently explored how pollination systems can be maintained from a demographic perspective by using population dynamics models (Holland and DeAngelis, 2010; Morris *et al.*, 2010; Valdovinos *et al.*, 2013; Ke and Nakazawa, 2018; Nakazawa and Kishi, 2023). These theoretical studies have typically assumed that plant–pollinator relationships are mutualistic, thereby focusing on preventing population explosion caused by the positive feedback between mutualistic species and subsequent extinction of other species by incorporating factors such as adaptive foraging, saturating benefits, and/or costs of mutualism (see references cited above).

However, it is notable that plant–pollinator relationships are not always mutualistic. Some flowering plants attract pollinating animals without providing any rewards, such as pollen and nectar (i.e. deceptive pollination). Their flowers attract potential pollinators by mimicking floral rewards, oviposition substrates and copulation mates (Dafni, 1984). Deceptive pollination is found in hundreds of genera in 32 families, which represents 4–6 % of all flowering plant species (Johnson and Schiestl, 2016). Nevertheless, it shows extreme diversification in several taxa (Okuyama and Kakishima, 2022) and thus provides suitable opportunities for studying the evolutionary stability of deceptive pollination (Jersáková *et al.*, 2006; Frederickson, 2013). Deceptive pollination causes pollinators to waste time on unrewarding flower visitation (de Jager and Ellis, 2014). In sexually deceptive pollination systems, where flowers mimic the copulation mates of pollinators, female pollinators may miss mating opportunities because male pollinators are attracted by floral decoys that emit odours similar to female sex pheromones (Wong and Schiestl, 2002; Wong *et al.*, 2004). In addition, sperm are sometimes wasted through fruitless copulation behaviour with deceptive plants (Gaskett *et al.*, 2008; Brunton Marin *et al.*, 2020). Therefore, deceptive pollination systems can be regarded as parasitic in contrast to mutualistic pollination systems where plants contribute to the prosperity of pollinators (Baguette *et al.*, 2020). This implies that the demographic vulnerability of deceptive pollination systems would be qualitatively different from that of general mutualistic pollination systems. At present, however, little effort has been made to elucidate the demographic vulnerability of deceptive pollination systems (but see Vázquez and Barradas, 2017; Brunton Martin *et al.*, 2021*b*).

**Fig. 1.**
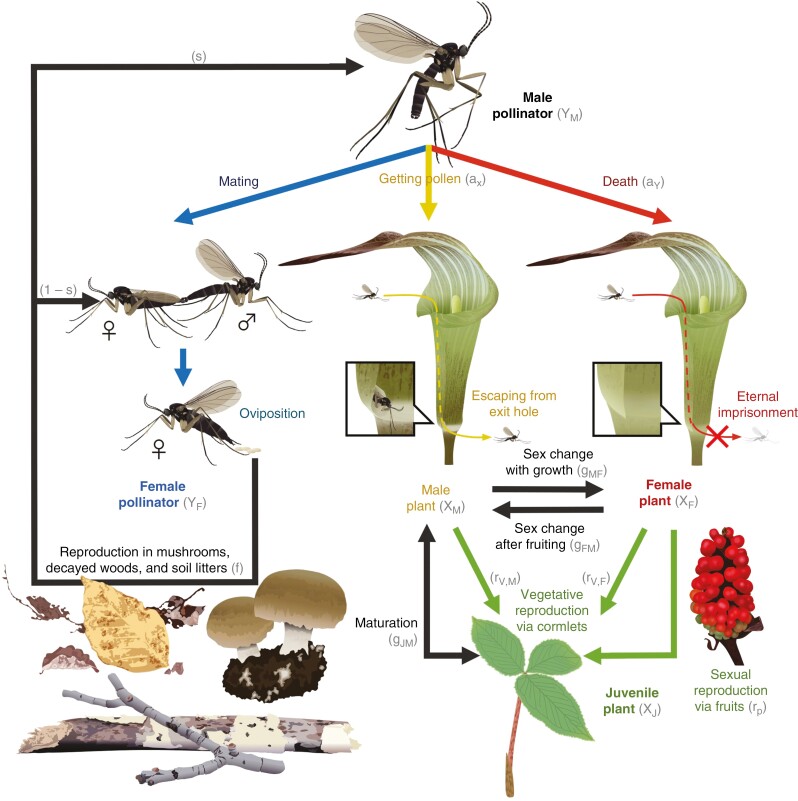
Conceptual illustration of the model for lethal deceptive pollination systems. The plant is male when it is small and becomes female as it grows and produces male plants via sexual reproduction when pollinated. Pollination occurs when male insects that have already visited male plants visit female plants. Meanwhile, both male and female plants produce male plants via vegetative reproduction. Both male and female plants are hypothesized to mimic female insects while only female plants trap male insects. Mating occurs when male insects successfully visit female insects. Female insects produce offspring with a specific sex ratio.

Here we focused on the population dynamics of a pollination system known as ‘lethal deceptive pollination’ in the plant genus *Arisaema* (Vogel and Martens, 2000; Suetsugu, 2022), because it represents the most extreme case of deceptive pollination systems. While *Arisaema* plants usually attract pollinating insects without providing any rewards as in other deceptive pollination systems (but see Suetsugu *et al.*, 2024), their unique aspect is that their pollinators are permanently trapped by the plants. In *Arisaema*, male plants have a gap in the spathe, which serves as an exit for pollinators, but female plants do not have it, thereby confining them. Eventually, the pollinators die within the female plants (Vogel and Martens, 2000; Suetsugu, 2022; Fig. 1). Some *Arisaema* species selectively attract male fungus gnats (Diptera: Mycetophilidae and Sciaridae) in a species-specific manner (Kakishima *et al.*, 2020; Matsumoto *et al.*, 2021, 2023*b*) using olfactory signals (Suetsugu *et al.*, 2021). Therefore, Suetsugu *et al.* (2021) hypothesized that the floral odour mimics female sex pheromones of fungus gnat pollinators. Although sexually deceptive pollination has been reported in other plant taxa in the families Asteraceae, Iridaceae and Orchidaceae (Bohman *et al.*, 2016), lethal imprisonment has not, to date, been identified, except in *Arisaema* (Suetsugu, 2022). Many questions remain regarding this pollination system. For example, the adaptive significance of confining pollinating insects remains poorly understood. Vogel and Martens (2000) presumed that imprisonment may prolong the pollinators’ contact with flowers, resulting in higher pollination success. Suetsugu (2022) speculated that confining pollinators may mitigate intraspecific competition among *Arisaema* seedlings by reducing the reproductive success of competing plants. From a demographic perspective, a natural question is, under what conditions can lethal deceptive pollination be maintained, given that pollinator exploitation by *Arisaema* is expected to reduce fungus gnat populations?

This question is worth asking because many *Arisaema* species are currently endangered in Japan (Murata *et al.*, 2018), the centre of species diversity for *Arisaema* (Murata, 1990). Many Japanese *Arisaema* species are endemic to narrow distributional areas, and one-third of them are included on the Japanese Red List (Murata *et al.*, 2018), probably due to various disturbances, such as reduction in forest habitat, modification of the forest understorey owing to increasing deer abundance, and plant theft for horticultural cultivation (Oshima *et al.*, 1997; Murata *et al.*, 2018; Matsumoto *et al.*, 2020). Different disturbance types may have different ecological consequences for *Arisaema* species. For example, forest habitat reduction negatively affects not only plant but also pollinator populations. Indeed, decreasing old forests or increasing agricultural lands and open coniferous woodlands can decrease abundance and species richness of fungus gnats in the vicinity (Økland, 1996; Økland *et al.*, 2005). Increasing deer abundance, which severely disturbs forest understorey habitats of *Arisaema* in temperate zones (Heckel *et al.*, 2010; Matsumoto *et al.*, 2020), may have positive and negative effects on plant and pollinator population sizes, respectively. In general, deer avoid feeding on *Arisaema* species (Hashimoto and Fujiki, 2014), owing to their high calcium oxalate crystal content (Bierzychudek, 1982). Therefore, such toxic plants are released from competition with other palatable plants as deer abundance increases (Augustine and McNaughton, 1998; Rooney and Waller, 2003). Meanwhile, fungus gnats rely on mushrooms, decayed wood, and soil litter in old forests for recruitment (Binns, 1981; Irmler *et al.*, 1996; Jakovlev, 2012), and are sensitive to forest understorey disturbances (Økland, 1994, 1996; Toft *et al.*, 2001). Therefore, soil degradation caused by abundant deer (Furusawa *et al.*, 2003; Heckel *et al.*, 2010; Fukushima *et al.*, 2014) may decrease fungus gnat populations, which, in turn, may negatively affect *Arisaema* species. Theft for horticultural cultivation has a direct negative effect on plant population size. Currently, it remains unclear how these different disturbance types mediate the population dynamics of *Arisaema* species.

In the present study, we aimed to theoretically investigate the population dynamics and disturbance responses of this lethal deceptive pollination system. We developed a mathematical model to describe the population dynamics of a deceptive plant and its pollinating insect victims. We also compiled existing data on life-history parameters and population abundance of multiple *Arisaema* species for parameterizing and calibrating the model. Thus, we attempted to provide the first general picture of lethal deceptive pollination systems from a demographic perspective and identify key parameters that can strongly influence their population dynamics and coexistence. In addition, using the model, we also explored how different types of disturbances can affect plant–insect coexistence in a lethal pollination system. Here, we manipulated parameters to represent reductions in forest habitat, increased deer abundance, and plant theft for horticultural cultivation. Based on these results, we emphasized the qualitatively distinct demographic vulnerability of lethal deceptive pollination systems compared with general mutualistic pollination systems.

## MATERIALS AND METHODS

### Biological assumptions for model development

We consider one deceptive plant species and its specialist pollinator, and assumed the following situation for describing their population dynamics (Fig. 1). The plant has a stage structure (i.e. immature juveniles and mature adults) and the adult stage has a sex structure (i.e. male and female plants). Juvenile plants are produced by two processes; one is vegetative reproduction (i.e. cormlet production) by both adult males and females, and the other is sexual reproduction (i.e. seed production through cross-pollination) by adult females (Bierzychudek, 1982). At the same time, we assumed that the recruitment success of juvenile plants is limited by open space available for colonization. Juvenile plants grow up to the adult male stage first and then switch to female (i.e. sex change) via body mass growth. Size-dependent sex changes are common in Japanese *Arisaema* species (Kinoshita, 1986). Developmental regression can also occur. Male plants may regress to the juvenile stage because of external factors such as physical damages and deterioration of environmental conditions (Lovett Doust and Cavers, 1982; Bierzychudek, 1984). Meanwhile, adult female plants may shrink and become males due to resource depletion, especially after sexual reproduction (Ohara, 2015).

We assumed that the pollinator also has a sex structure (i.e. male and female insects). Because both male and female plants are hypothesized to attract the insect for pollination by releasing odours similar to the sex pheromones of female insects, only male insects contribute to pollination success of the plant (Suetsugu *et al.*, 2021; Matsumoto *et al.*, 2023*b*). Pollination of the plant is achieved when male insects collect pollen at male plants and thereafter visit a female plant. Attracted male insects are imprisoned because the inner surface of the spathe tube is covered by a slippery wax layer that prevents them from escaping (Vogel and Martens, 2000). Male plants have an exit hole at their base of their spathe tubes that allows male insects to escape, whereas female plants do not (Fig. 1). Therefore, the pollinating insects die within the spathe tube of female plants due to eternal imprisonment (Vogel and Martens, 2000; Suetsugu, 2022). Insect reproduction occurs when male insects visit female insects, and offspring production may increase with increasing copulations by male insects. Both pollination success of the plant and mating success of the pollinating insect would increase but saturate with visits by male insects because reproductive resources are limited.

### Model development

We developed a mathematical model to describe the population dynamics of lethal deceptive pollination systems as described above (Fig. 1; see Table 1 for parameter definitions). The model can be formulated as follows:

**Table 1.** Parameter definitions and default values.

	Definition	Default value
Variable		
*X*_*J*_*(t), X*_*M*_*(t), X*_*F*_*(t)*	Plant abundance (immature, adult male and adult female)	–
*Y_M_(t), Y_F_(t)*	Male and female insect abundance	–
Function		
*Q*_1_	Expected number of male plants a male insect visits before he dies at a female plant	–
*Q*_2_	Expected number of male insects copulating with a female insect	–
*P*	Probability that a male insect visits a female plant during lifespan	–
Parameter		
*a*_M_, *a*_F_	Attractiveness of male and female plants to male insects	Varied
*r*_V,M,_*r*_V,F_	Vegetative growth rate due to cormlet production by male and female plants	Varied
*r*_P_	Sexual reproduction rate due to pollination	1
*K*_*X*_, *K*_*Y*_	Recruitment capacity of plant and insect	15
*g*_JM_, *g*_MF_, *g*_FM_	Rates of transition from juvenile to male stage (net maturation), from male to female stage (sex change), and from female to male stage (sex reversal)	0.05, varied, 0.01
*d*_J_, *d*_M_, *d*_F_	Death rates of plant (immature, adult male and adult female)	0.015, 0.01, 0.01
*s*	Ratio of born male of insect	0.5
*f*	Female offspring production of insect	2
*dY*	Death rate of insect	0.1
*A*_*X*_, *A*_*Y*_,	Saturation coefficients in pollination and mating success	1, 10
*q*	Coefficient scaling lifetime probability of visiting a female plant to insect death rate	0.5
*N*	Maximum number of visits made by a male insect to plants or female insects	10


$$\begin{array}{l} \frac{dX_{J}}{dt}=\left[r_{V,\;M}X_{M}+\left(r_{V,F}+r_{P}\frac{Y_{M}Q_{1}}{A_{X}+Y_{M}Q_{1}}\right)X_{F}\right]\;\left(1-\frac{X_{J}+X_{M}+X_{F}}{K_{X}}\right)-g_{JM}X_{J}-d_{J}X_{J} \end{array}$$ (1a)

$$\begin{array}{l} \frac{dX_{M}}{dt}=g_{JM}X_{J}-g_{MF}X_{M}+g_{FM}\frac{Y_{M}Q_{1}}{A_{X}+Y_{M}Q_{1}}X_{F}-d_{M}X_{M} \end{array}$$ (1b)

$$\begin{array}{l} \frac{dX_{F}}{dt}=g_{MF}X_{M}-g_{FM}\frac{Y_{M}Q_{1}}{A_{X}+Y_{M}Q_{1}}X_{F}-d_{F}X_{F} \end{array}$$ (1c)

$$\begin{array}{l} \frac{dY_{M}}{dt}=sf\frac{Y_{M}Q_{2}}{A_{Y}+Y_{M}Q_{2}}\left(1-\frac{Y_{M}+Y_{F}}{K_{Y}}\right)Y_{F}-\left(d_{Y}+qP\right)Y_{M} \end{array}$$ (1d)

$$\begin{array}{l} \frac{dY_{F}}{dt}=\left(1-s\right)\;f\frac{Y_{M}Q_{2}}{A_{Y}+Y_{M}Q_{2}}\left(1-\frac{Y_{M}+Y_{F}}{K_{Y}}\right)Y_{F}-d_{Y}Y_{F} \end{array}$$ (1e)

where *X*_*i*_ (*i* = *J*, *M* or *F*) is plant abundance and *Y*_*j*_ (*j* = *M* or *F*) is insect abundance. The subscripts *J*, *M* and *F* represent juvenile, male and female plants, respectively. The model describes the population dynamics of the plant at the flower scale because the plant produces only a single inflorescence per year (Matsumoto *et al.*, 2024) and each inflorescence (i.e. spadix) of aroids generally functions as a single flower during the pollination process (Méndez, 2001). That is, one plant individual corresponds to one flower. We used ordinary differential equations for analytical tractability. The model included neither environmental fluctuation nor individual variation to simplify the model structure. The functional forms of biotic interaction terms (i.e. plant pollination and insect mating) followed Holling’s type II functional responses. The abundance of juvenile plants (*X*_*J*_) increases due to cormlet production (*r*_*V,i*_) by the male (*X*_*M*_) and female plants (*X*_*Y*_) and successful pollination (*r*_*P*_) (eqn 1a). The parameters *g*_*JM*_, *g*_*MF*_, and *g*_*FM*_ determine the rates of size-dependent transition from juvenile to adult male (i.e. maturation), from male to female stage (i.e. sex change) and from female to male (i.e. sex reversal following sexual reproduction), respectively (eqn 1a–c). We note that the transition rate *g*_*JM*_ encompasses the regression from male to juvenile stage, considering that it occurs randomly due to external events such as physical damage and deterioration of environmental conditions (Lovett Doust and Cavers, 1982; Bierzychudek, 1984). That is, the parameter biologically represents the net maturation rate (eqn 1a and b). When the parameter is low, it means that male plants may frequently regress to the juvenile stage. Meanwhile, we assumed that transition from female to male stage *g*_*FM*_ is proportional mainly to pollination success (eqn 1b and c). The pollination success of female plants ($\frac{Y_{M}Q_{1}}{A_{X}+Y_{M}Q_{1}}$) increases with the number of male plants insects visit before they die within female flowers *Q*_1_ (eqn 1a); however, it promotes sex reversal from female to male (eqn 1b and c). Similarly, the mating success of female insects ($\frac{Y_{M}Q_{2}}{A_{Y}+Y_{M}Q_{2}}$) increases with the number of copulations by male insects before they are trapped by female flowers *Q*_2_ (eqn 1d and e). Thereafter, female insects reproduce male and female offspring (*f*) at a ratio of *s* to 1 − *s*. We assumed *s* = 0.5 in the model analysis (Table 1), considering that the secondary sex ratio is generally 1:1 in sciarid populations under moderate temperate conditions (Sasakawa and Akamatsu, 1978; Nigro *et al.*, 2007; Shirvani Farsani *et al.*, 2013). Pollination and mating success increase but become saturated with the abundance of male insects, and *A*_*X*_ (eqn 1a) and *A*_*Y*_ (eqn 1d and e) determine the saturation rates, respectively.

It is also assumed that the population growth of plants and insects is limited by *K*_*X*_ (eqn 1a) and *K*_*Y*_ (eqn 1d and e), respectively. We refer to this as recruitment capacity. The parameter *K*_*X*_ represents the area of habitat that the plant can inhabit, which can be affected by forest habitat reduction and the reduction in competitive plants under deer herbivory. It is likely that *K*_*X*_ will decrease with forest habitat reduction but increase with increasing deer abundance (Oshima *et al.*, 1997; Matsumoto *et al.*, 2020). The parameter *K*_*Y*_ represents the availability of brood sites for insects, such as mushrooms, decayed wood, and soil litter (Binns, 1981; Irmler *et al.*, 1996; Jakovlev, 2012). We assume that *K*_*Y*_ would decrease with forest habitat reduction and increasing deer abundance. The plant has stage-specific death rates, *d*_*J*_, *d*_*M*_ and *d*_*F*_ (Bierzychudek, 1982; Kinoshita, 1987), which are expected to increase with theft for horticultural cultivation (Fukai, 2007; Naito *et al.*, 2015; Murata *et al.*, 2018). As default values, we assumed that the death rate of juvenile plants is higher than that of adult plants while male and female adult plants have the same death rate (Table 1). This was because the survival rate increases with body mass but is saturated when plant size exceeds the threshold of flowering (Bierzychudek, 1982; Kinoshita, 1987). Male and female insects have a natural death rate *d*_*Y*_, but only male insects have an additional mortality term owing to visiting female plants (eqn 1d and e). This is proportional to the lifetime probability of visiting a female plant (*P*). The parameter *q* is introduced to unify the scale of the lifetime trap probability *P* with that of the per-unit time death rate. The default values of parameters are provided in Table 1.

The relative attractiveness of flowers and female insects to male insects is a key parameter in the model. We assume that these processes are regulated by parameters *a*_*M*_ and *a*_*F*_ as follows:


$$\begin{array}{l} p_{M}=\frac{a_{M}X_{M}}{a_{M}X_{M}+a_{F}X_{F}+Y_{F}} \end{array}$$ (2a)

$$\begin{array}{l} p_{F}=\frac{a_{F}X_{F}}{a_{M}X_{M}+a_{F}X_{F}+Y_{F}} \end{array}$$ (2b)

$$\begin{array}{l} p_{Y}=\frac{Y_{F}}{a_{M}X_{M}+a_{F}X_{F}+Y_{F}} \end{array}$$ (2c)

where *p*_*M*_, *p*_*F*_, and *p*_*Y*_ are the probabilities of visiting a male plant, female plant or female insect per visit event, respectively (i.e. *p*_*M*_ + *p*_*F*_ + *p*_*Y*_ = 1). When parameters *a*_*M*_ and *a*_*F*_ are zero, male insects always visit female insects in preference to deceptive plants, and their probability of successful mating (i.e. *p*_*Y*_) decreases as these parameters increase (eqn 2c). When the parameters are 1 (i.e. *a*_*M*_ = *a*_*F*_ = 1), male insects visit deceptive plants or female insects without discrimination, that is, at random, depending on the relative abundance of deceptive plants (eqn 2a and b) and female insects (eqn 2c). These parameters can be defined as the floral attractiveness of deceptive plants relative to that of female insects.

Male insects have multiple opportunities to visit deceptive plants or female insects during their lifetime, which can affect their overall pollination and mating success. Therefore, the expected number of visits to male plants or female insects (*Q*_1_, *Q*_2_, and *P*) depends on the number of *k* combinations from *j* visiting events (_*j*_*C*_*k*_) and its probability *p*_*i*_ (eqn 2a–c) as follows (see Supplementary Data Information S1 for detailed derivations):


$$\begin{array}{l} Q_{1}=p_{F}\sum_{j=0}^{N-1}\sum_{k=0}^{\;j}k{(}_{j}C_{k}){\left(p_{M}\right)}^{k}{\left(p_{Y}\right)}^{\;j-k} \end{array}$$ (3a)

$$\begin{array}{lll} Q_{2}= & p_{F}\sum_{j=0}^{N-1}\sum_{k=0}^{\;j}k{(}_{j}C_{k}){\left(p_{Y}\right)}^{k}{\left(p_{M}\right)}^{\;j-k}\; & +\sum_{k=0}^{N}k{(}_{N}C_{k}){\left(p_{Y}\right)}^{k}{\left(p_{M}\right)}^{N-k} \end{array}$$ (3b)

$$\begin{array}{l} P=p_{F}\sum_{k=0}^{N-1}{\left(1-p_{F}\right)}^{k-1} \end{array}$$ (3c)

where the parameter *N* is the lifetime number of visiting events in relation to the lifespan of male insects. The summation term in *Q*_1_ gives the expected number of male plants a male insect visits until he finally dies at a female plant (eqn 3a). The summation term in the first term of *Q*_2_ gives the expected number of female insects a male insect visits before finally dying at a female plant (eqn 3b). The second summation of *Q*_2_ gives the expected number of female insects a male insect visits without visiting a female plant (eqn 3b). *P* is the expected probability that a male insect visits and dies at a female plant during its lifetime (eqn 3c).

### Model analysis

First, we mathematically analysed the persistence of the plant population. Persistence is defined as the potential of the plant population to recover when both plants and insects are rare. In the absence of insects, plant population dynamics can be described as follows.


$$\begin{array}{lll} \frac{dX_{J}}{dt}= & \left(r_{V,M}X_{M}+r_{V,F}X_{F}\right)\left(1-\frac{X_{J}+X_{M}+X_{F}}{K_{F}}\right)\; & -g_{JM}X_{J}-d_{J}X_{J} \end{array}$$ (4a)

$$\begin{array}{l} \frac{dX_{M}}{dt}=g_{JM}X_{J}-g_{MF}X_{M}-d_{M}X_{M} \end{array}$$ (4b)

$$\begin{array}{l} \frac{dX_{F}}{dt}=g_{MF}X_{M}-d_{F}X_{F} \end{array}$$ (4c)

The plant population is persistent if:


$$\begin{array}{l} \frac{g_{JM}}{g_{JM}+d_{J}}\left(\frac{r_{V,M}}{g_{MF}+d_{M}}+\frac{g_{MF}}{g_{MF}+d_{M}}\frac{r_{V,F}}{d_{F}}\right)>1 \end{array}$$ (5)

This is the product of the expected probability of a juvenile plant surviving to the adult male stage (the first term) and the expected number of juvenile plants produced by the adult plants (the sum in the second term) (eqn 5; see Schreiber and Rudolf, 2008; Ke and Nakazawa, 2018 for derivations of similar formulations). In the sum term, the first term is the expected number of juvenile plants produced by one male plant during the survival period and the second term is the product of the probability of a male plant surviving to the female stage and the expected number of juvenile plants produced by one female plant during the survival period. The plant population persists when the lifetime fitness represented by the left side is greater than 1 (eqn 5). This inequality illustrates that the plant cannot invade the trivial equilibrium (*X*_*i*_ = *Y*_*i*_ = 0), i.e. the plant cannot exist alone without the insect when the rates of vegetative reproduction (*r*_*VM*_ and *r*_*VF*_), transition from juvenile to male (*g*_*JM*_) and transition from male to female (*g*_*MF*_) are low.

Next, we numerically simulated plant–insect population dynamics. We manipulated not only the floral attractiveness but also the recruitment capacity of both plants (*K*_*X*_) and insects (*K*_*Y*_) and the death rates of the adult plants (*d*_*M*_ and *d*_*F*_) to assess the effects of different types of disturbances, namely forest habitat reduction, increased deer abundance, and theft for horticultural cultivation on plant–insect population dynamics. To determine other parameter values, we experimented with different values within reasonable ranges so that the simulated sex ratio was in accordance with the empirical data (see below). To verify the reliability and improve the robustness of results, we performed a sensitivity analysis in which we varied one parameter value while fixing the others at default values. For all the numerical simulations, the initial population abundances were *X*_*J*_(0) = 1, *X*_*M*_(0) = *X*_*F*_(0) = 0 and *Y*_*M*_(0) = *Y*_*F*_(0) = *K*_*Y*_/2. The initial conditions resembled the situation in which juvenile plants colonized a plant-free steady state. We then simulated the model until the dynamics converged to the steady state (*t* = 50 000) and evaluated the long-term average abundance of plants and insects during the last 10 % of the period (*t* = 45 000–50 000), considering the possibility that the steady-state dynamics may fluctuate due to their parasitic relationship (see Introduction). The threshold population size for extinction was set to 0.001. All the numerical simulations were conducted using NDSolve in Mathematica version 12.

### Empirical data for model parameterization and calibration

We also collected empirical data to calibrate the parameters and validate the predictions of the developed model. To this end, we extracted published data from previous studies (Matsumoto *et al.*, 2020, 2021; Matsumoto, 2021) on four Japanese *Arisaema* species (*A. ovale*, *A. angustatum*, *A. peninsulae* and *A. pseudoangustatum* var. *pseudoangustatum*) attracting species-specific pollinators in sympatric populations (see Matsumoto *et al.*, 2020, 2021; Matsumoto, 2021 for detailed methodology) while obtaining additional data for the present study. All the statistical analyses below were conducted using R software version 3.5.1 (R Development Core Team, 2018).

#### Threshold plant size of flowering (transition from juvenile to adult male)

In our model, the parameter *g*_*JM*_ determines the rate of transition from juvenile to male adult. To examine the interspecific difference in the maturation rate, we re-analysed the plant size data of *A. peninsulae* (male: *n* = 10; juvenile: *n* = 42) and *A. ovale* (male: *n* = 72; juvenile: *n* = 1054), adopted from Matsumoto *et al.* (2020). We estimated the size-dependency of flowering by constructing a generalized linear model (GLM) with binomial error distribution and logit link function using function glm in R. We considered maturation state (juvenile = 0, flowering = 1) and species (*A. peninsulae* or *A. ovale*) as the response variables and pseudostem diameter, i.e. a proxy of plant biomass (Kinoshita, 1986), as the explanatory variable. We determined the significance of the estimated parameters using a Wald type II *χ*^2^ test. We regarded the estimated inflection point as the threshold plant size for flowering (Heckel and Kalisz, 2017).

#### Threshold plant size of sex change from male to female

We also examined whether adult plants have species-specific thresholds to change sex expression from male to female. A lower threshold size corresponds to higher rates of transition *g*_*MF*_ in the model. To test interspecific differences in the threshold size, we re-analysed the plant size data provided by Matsumoto (2021) for *A. angustatum* (female: *n* = 10, male: *n* = 37) and *A. pseudoangustatum* var. *pseudoangustatum* (female: *n* = 7; male: *n* = 48). In addition, the plant size data for the other two species were obtained in the present study using the same method: *A. ovale* (female: *n* = 13; male: *n* = 45) and *A. peninsulae* (female: *n* = 14; male: *n* = 28). Similar to the above analysis, we constructed GLMs for each species. We considered sex expression (male = 0, female = 1) as the response variable and pseudostem diameter as the explanatory variable. To examine the interspecific differences in threshold plant size, we conducted multiple comparisons using logistic regression analyses for all six species pairs. We adjusted the *P*-values of Wald type II *χ*^2^ tests for the estimated coefficient of the ‘species’ factor using Holm’s methods.

#### Vegetative reproduction

Cormlet productivity was used as a proxy for the vegetative reproduction rate *r*_*V*_. Matsumoto *et al.* (2020) provided data for *A. ovale* (female: *n* = 3; male: *n* = 7; juvenile: *n* = 40) and *A. peninsulae* (female: *n* = 2; male: *n* = 5; juvenile: *n* = 8). Cormlet productivity was not examined in the other two species (*A. angustatum* and *A. pseudoangustatum* var. *pseudoangustatum*) but they rarely exhibit vegetative reproduction under cultivated conditions (T. K. Matsumoto, personal observations).

#### Sex-specific floral attractiveness

We used the visitation frequency of pollinators (fungus gnats) as a proxy for the sex-specific floral attractiveness of each species. It was not easy to compare floral attractiveness among different *Arisaema* species based only on pollinator visitation frequency because different plant species rely on different pollinator species (Kakishima *et al.*, 2020; Matsumoto *et al.*, 2021, 2023*b*; Suetsugu *et al.*, 2021) and the actual population abundance of each pollinator species in natural habitats is unknown. Matsumoto *et al.* (2021) provided data on visitation frequency by species-specific pollinators of the four *Arisaema* species: *A. ovale* (female: *n* = 16; male: *n* = 51), *A. angustatum* (female: *n* = 9; male: *n* = 44), *A. peninsulae* (female: *n* = 13; male: *n* = 31) and *A. pseudoangustatum* var. *pseudoangustatum* (female: *n* = 9; male: *n* = 49). To examine intersexual difference in floral attractiveness, we constructed GLMs with negative binomial error distribution and log link function using the glm.nb function in the MASS package (Ripley *et al.*, 2022) in R. GLMs were conducted separately for each species. We considered the number of species-specific pollinators captured on each plant during the whole flowering season as a response variable and the sex expression of plants as explanatory variables. We determined the significance of the estimated parameters using a Wald type II *χ*^2^ test.

#### Population abundance and sex ratio

Matsumoto *et al.* (2021) measured the abundances of male and female plants of the four *Arisaema* species in sympatric populations: *A. ovale* (female: *n* = 144; male: *n* = 372), *A. angustatum* (female: *n* = 28; male: *n* = 66), *A. peninsulae* (female: *n* = 28; male: *n* = 89) and *A. pseudoangustatum* var. *pseudoangustatum* (female: *n* = 20, male: *n* = 90). To examine the interspecific difference in sex ratio, we conducted multiple comparisons by *χ*^2^ tests for all six species pairs using the function chisq.test in R. The *P*-values were adjusted using Holm’s method. The sex-ratio data will be used to calibrate the model.

## RESULTS

**Fig. 2.**
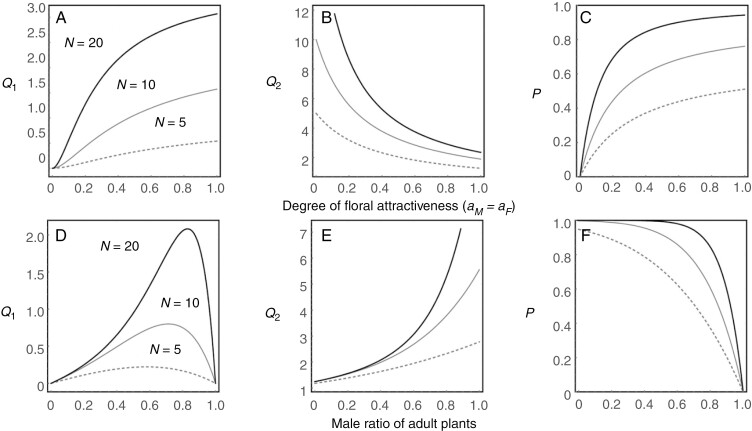
Effects of floral attractiveness and plant sex ratio on visiting behaviour of male insects. The upper panels show the effects of the sex-generic degree of floral attractiveness (*a*_*M*_ = *a*_*F*_) on (A) the number of male plants one male insect visits before he dies at a female plant (*Q*_1_), (B) the number of female insects one male insect visits before he dies at a female plant (*Q*_2_), and (C) the expected probability that one male insect visits and dies at a female plant during his lifetime (*P*). For presentation purposes, the male ratio of the plant and insect is fixed at 0.8 and 0.5, respectively. The sex ratio of the plant is male-biased based on field observations (Fig. 3D). The bottom panels show the effects of male ratio of the plant on (D) *Q*_1_, (E) *Q*_2_ and (F) *P*. For presentation purposes, the sex-generic floral attractiveness is fixed at *a*_*M*_ = *a*_*M*_ = 0.4 and the abundance of female insects is fixed at *Y*_*F*_ = *K*_*Y*_/2. The dashed, solid and thick lines indicate *n* = 5, 10, and 20, respectively.

We observed that *Q*_1_ and *P* monotonically increase and *Q*_2_ monotonically decreases with floral attractiveness (Fig. 2A–C). These patterns indicate that floral attractiveness improves plant pollination success (Fig. 2A), thereby suppressing insect mating success (Fig. 2B), and increases insect mortality (Fig. 2C). We also found that *Q*_1_ varies unimodally, *Q*_2_ increases monotonically and *P* decreases monotonically with the plant male sex ratio (Fig. 2D–F). These patterns indicate that plant pollination success is maximized at a male-biased sex ratio (0.6–0.8; Fig. 2D), and male dominance in the plant population improves the mating success of the insects (Fig. 2E) while decreasing their mortality (Fig. 2F).

**Fig. 3.**
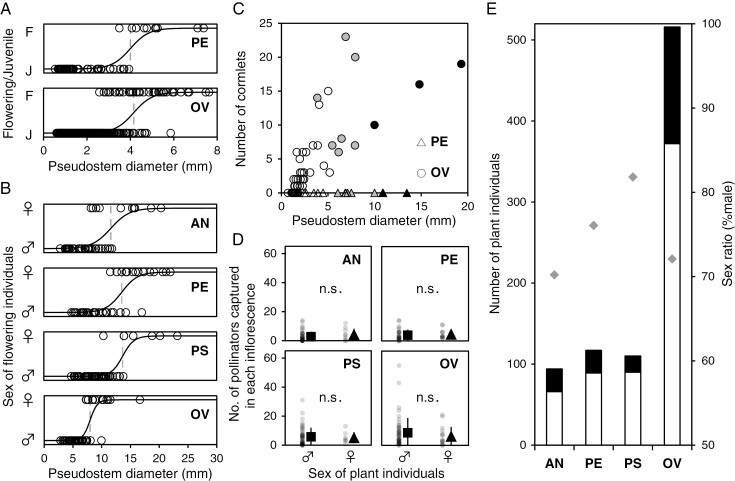
Field observation data of four sympatric *Arisaema* species. (A) Threshold plant size of flowering (maturation from juvenile to adult male) in two *Arisaema* species. (B) Threshold plant size of sex change from male to female. In panels (A) and (B), the solid and broken lines show the logistic regressions and estimated inflection points, respectively. (C) Relationship between plant size and vegetative reproduction (number of cormlets) in two *Arisaema* species. White, grey and black symbols indicate juveniles, males and females, respectively. (D) Visitation frequency of pollinators to male and female plants. (E) Population abundance and sex ratio. White and black columns indicate the numbers of male and female plants and grey diamonds indicate the sex ratio in each species. Abbreviations of species names are OV, *Arisaema ovale*; AN, *A. angustatum*; PE, *A. peninsulae*; and PS, *A. pseudoangustatum* var. *pseudoangustatum*.

The empirical data showed a significant correlation between maturation state and plant size in both *A. peninsulae* and *A. ovale* (*P* < 0.001), but no difference in threshold size was detected between the two species (*P* = 0.58) (Fig. 3A). While significant correlations between sex expression and plant size were detected in all species (Fig. 3B), the threshold plant size for sex change was significantly lower in *A. ovale* than in the other three species (*P* < 0.05). No significant differences were detected among the other three species (*P* > 0.05; Fig. 3B). Cormlet productivity was obviously higher in *A. ovale* than in *A. peninsulae* (Fig. 3C), as reported in previous studies (Murata, 1993; Murata *et al.*, 2018). The cormlet productivity of adult plants (mean ± standard deviation) was approximately five times higher than that of juveniles in *A. ovale* (13.6 ± 6.2 versus 2.6 ± 3.4) owing to larger plant biomass, while the size dependency was not very clear among adult plants (Takasu, 1988; Heckel and Kalisz, 2017; T. K. Matsumoto, unpubl. data; Fig. 3C). Moreover, 14 of 40 juveniles in *A. ovale* did not produce any cormlets (Fig. 3C) while the viability of cormlets produced by tiny juveniles appeared to be very low (T. K. Matsumoto, personal observation). This supports the model assumption that juvenile plants lack cormlet productivity. The visitation frequency of fungus gnats was not significantly different between male and female plants for all four *Arisaema* species (Fig. 3D).

Based on the above empirical data, we manipulated or parameterized the model as follows. Notably, we identified the two main cases for model analysis. In the first case (hereafter the first scenario), the rates of vegetative reproduction and transition from male plant to female plant were low, which applied to *A. angustatum*, *A. peninsulae* and *A. pseudoangustatum* var. *pseudoangustatum* (Fig. 3B, C). In the second case (hereafter the second scenario), these parameters were both high, which applied to *A. ovale* (Fig. 3B, C). Therefore, we manipulated these parameters for scenario-based analysis of the demographic consequences of floral attractiveness and disturbance impact, while assuming that the rate of vegetative reproduction was the same between male and female plants (i.e. *r*_*V,M*_ = *r*_*V,F*_) (Fig. 3C). Although the transition rate from male to female differed between the two scenarios (Fig. 3B), we assumed the same transition rate from juvenile to male between them (Fig. 3A). Further, we manipulated floral attractiveness while maintaining consistency between male and female plants (i.e. *a*_*M*_ = *a*_*F*_) in both scenarios (Fig. 3D). While *A. ovale* was more abundant than the other three species (Fig. 3E), the sex ratio was equally male-biased (0.7–0.8) for all four species. Therefore, we calibrated the model so that the male ratio was close to 0.7 at the steady state in both the first and second scenarios. Through manual parameter exploration within reasonable ranges, the default parameter values were set as *r*_*P*_ = 1, *d*_*J*_ = 0.015, *d*_*M*_ = *d*_*F*_ = 0.01, *d*_*Y*_ = 0.1, *g*_*JM*_ = 0.05, *g*_*FM*_ = 0.01, *s* = 0.5, *f* = 2, *q* = 0.5, *A*_*X*_ = 1, *A*_*Y*_ = 10 and *N* = 10 (Table 1). We used these values in the following model analysis (see Supplementary Data Information S2 for sensitivity analysis).

**Fig. 4.**
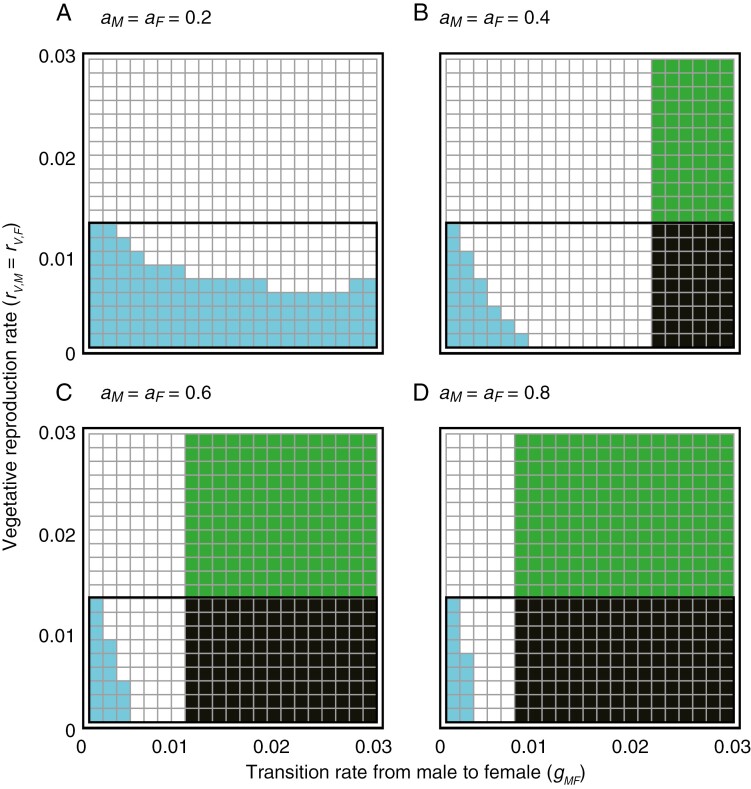
Effects of rates of transition from male to female and vegetative reproduction on plant–insect population dynamics. Different colours indicate the steady state of the population dynamics. The plant and insect coexist in white cells, only the plant exists in green cells, only the insect exists in blue cells, and both go extinct in black cells. The solid line indicates the persistence condition of the plant under which the plant cannot persist alone. The sex-generic floral attractiveness (*a*_*M*_ = *a*_*F*_) is (A) 0.2, (B) 0.4, (C) 0.6 and (D) 0.8. Other parameters are given in Table 1.

We found that plant–insect coexistence critically depends on the rates of both vegetative reproduction (*r*_*VM*_ =* r*_*VF*_) and transition rate from male to female (*g*_*MF*_) (Fig. 4). For low levels of floral attractiveness (*a*_*M*_ = *a*_*F*_ = 0.2), they are more likely to coexist when the vegetative reproduction rate is high (white cells in Fig. 4A), whereas the plant becomes extinct when the rate of vegetative reproduction is low (blue cells in Fig. 4A). The plant population can coexist with the insect even when it cannot persist alone because of low vegetative reproduction rates (white cells below the solid line in Fig. 4A). In this parameter region, sexual reproduction mediated by pollinators causes alternative stable states in which the plant goes extinct or coexists with the insect, depending on the initial conditions.

The effects of the transition rate from male to female become apparent when floral attractiveness is higher (Fig. 4). With increasing floral attractiveness, the likelihood of plant extinction due to low transition rate from male to female is mitigated (see the shrinkage of blue cells from Fig. 4A to D). Meanwhile, the insect population becomes more likely to go extinct (see the expansion of green cells from Fig. 4A to D). Similarly, the likelihood of co-extinction increases with floral attractiveness (see the expansion of black cells from Fig. 4A to D). These results emerge when the transition rate from male to female (*g*_*MF*_) is high. In this parameter space, female plants increase pollination success but reduce the pollinating insects. Once the insect population collapses, the plant also becomes extinct if the vegetative reproduction rate is low (black cells in Fig. 4B–D). Otherwise, the plant survives through vegetative reproduction but the insect does not survive (green cells in Fig. 4B–D). Consequently, plants with high floral attractiveness can coexist with insects only when the transition rate from male to female is moderately low (white cells in Fig. 4B–D). The sensitivity analysis confirmed that our results are generally robust to changes in parameters (results and discussion on the sensitivity analysis are provided in Supplementary Data Information S2). We also found that the plant tends to be highly male-biased when it coexists with the insect (Supplementary Data Fig. S1). The male-biased sex ratio in the coexistence region is consistent with our field observational data (Fig. 3D), suggesting that plant sex ratio would be crucial for plant–insect coexistence.

**Fig. 5.**
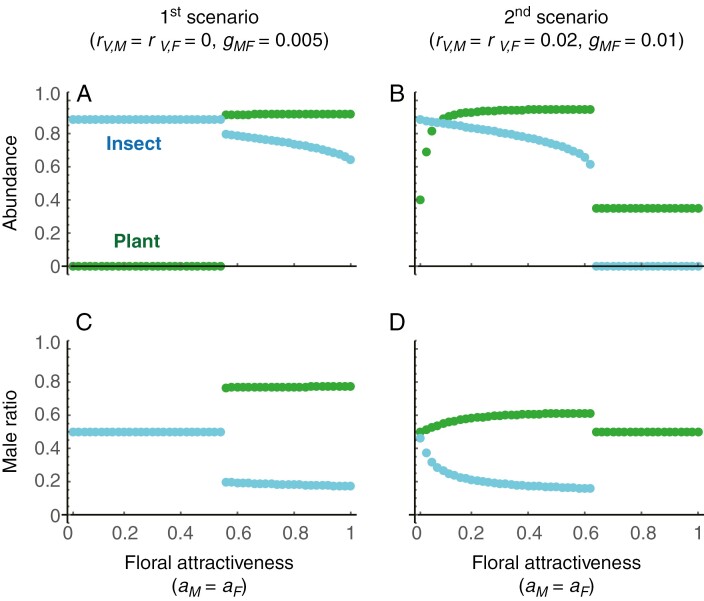
Effects of floral attractiveness on population abundance and sex ratio of the plant and insect. Vegetative reproduction is absent (*r*_*V,M*_ = *r*_*V,F*_ = 0) and the rate of transition from male to female is low (*g_MF_* = 0.005) in the left panels. Vegetative reproduction is present (*r*_*V,M*_ = *r*_*V,F*_ = 0.02) and the rate of transition from male to female is relatively high (*g* = 0.01) in the right panels. (A, B) Population abundances of plants and insects relative to *K*_*X*_ or *K*_*Y*_. (C, D) Male ratios of plants and insects. The green and blue lines indicate the plant and insect, respectively. In each panel the *x*-axis represents sex-generic floral attractiveness (*a*_*M*_ = *a*_*F*_). Other parameters are provided in Table 1.

We compared the effects of floral attractiveness on plant–insect population dynamics between the two main scenarios identified based on empirical data (Fig. 3B, C). In the first scenario, where vegetative reproduction is absent (*r*_*VM*_ = *r*_*VF*_ = 0) and the transition rate from male to female is low (*g*_*MF*_ = 0.005), the plant is likely to coexist with the insect when floral attractiveness is high (Fig. 5A). This is because plant population growth is diminished when floral attractiveness is low. In the second scenario, where vegetative reproduction is present (*r*_*VM*_ = *r*_*VF*_ = 0.02) and the transition rate from male to female is high (*g*_*MF*_ = 0.01), the plant is only reduced in abundance and rarely goes extinct, even when the insect goes extinct owing to high floral attractiveness (Fig. 5B). This is because the plant population persist through vegetative reproduction. The difference in plant extinction potential between the two scenarios may explain the difference in abundance between *A. ovale* with high vegetative reproduction ability and the three other species lacking it (Fig. 3E). The sex ratio of the plant is also male-biased (Fig. 5C, D), which is consistent with the empirical data (Fig. 3E) because of parameter calibration. The sex ratio of the insects is close to 0.5, when plant abundance is low, but becomes female-biased as floral attractiveness increases (Fig. 5C, D). This is because male insects are trapped by the female plants.

**Fig. 6.**
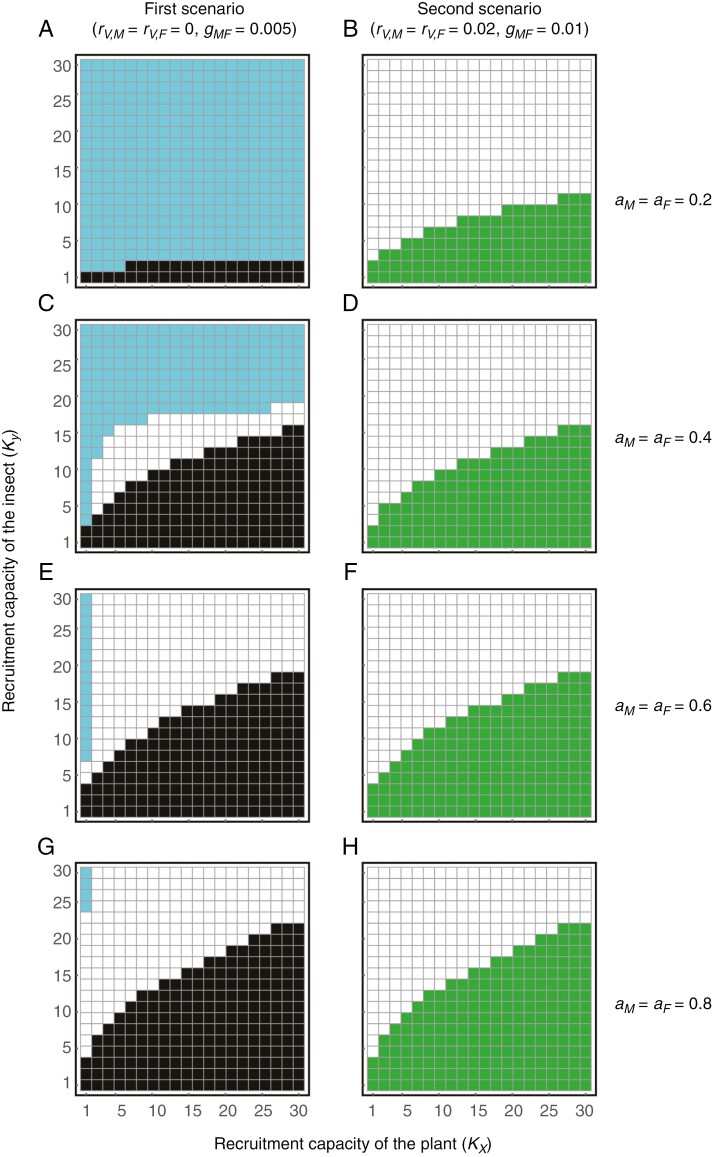
Effects of recruitment capacity of the plant and insect on plant–insect population dynamics. In the left panels vegetative reproduction is absent (*r*_*V,M*_ = *r*_*V,F*_ = 0) and the rate of transition from male to female is low (*g_MF_* = 0.005). In the right panels vegetative reproduction is present (*r*_*V,M*_ = *r*_*V,F*_ = 0.02) and the rate of transition from male to female is high (*g_MF_* = 0.01). Different colours indicate the steady state of the population dynamics. In each panel the *x*- and *y*-axes represent the recruitment capacity of plants and insects, respectively. The plant and insect coexist in white cells, only the plant exists in green cells, only the insect exists in blue cells, and both go extinct in black cells. The sex-generic floral attractiveness (*a*_*M*_ = *a*_*F*_) is (A, B) 0.2, (C, D) 0.4, (E, F) 0.6 and (G, H) 0.8. Other parameters are presented in Table 1. The effects of forest habitat reduction can be described by a decrease in both *K*_*X*_ and *K*_*Y*_, whereas the effects of increasing deer herbivory can be described by an increase in *K*_*X*_ and decrease in *K*_*Y*_.

**Fig. 7.**
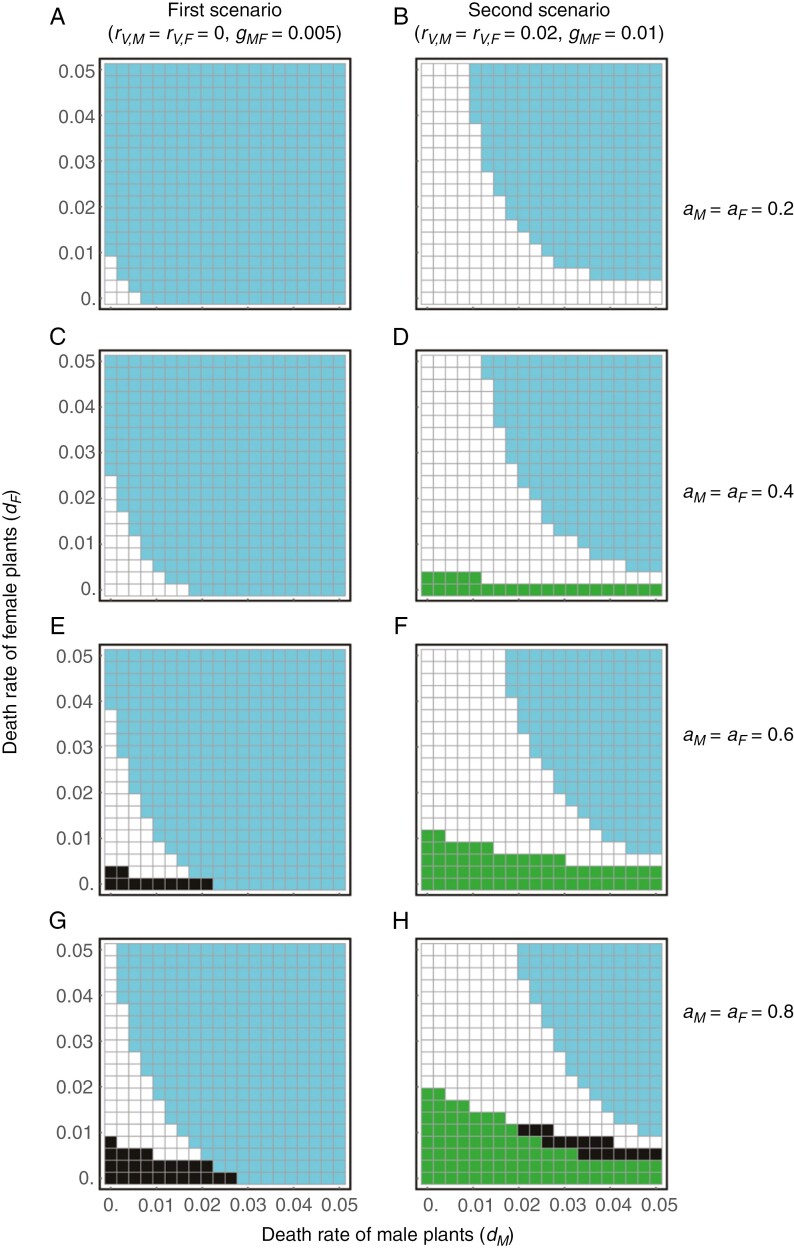
Effects of sex-specific death rates of the plant on plant–insect population dynamics. In each panel the *x*- and *y*-axes represent the death rate of male and female plants, respectively. The effects of plant theft can be described by an increase in *d*_*M*_ and *d*_*F*_. Other notations are the same as those in Fig. 6.

We found that manipulations of *K*_*X*_, *K*_*Y*_, *d*_*M*_ and *d*_*F*_ (i.e. disturbances) can have distinct consequences on plant–insect population dynamics depending on the scenario (Figs 6 and 7). In the first scenario (i.e. *r*_*VM*_ = *r*_*VF*_ = 0 and *g*_*MF*_ is low), the plant tends to become extinct when it has a low recruitment capacity, *K*_*X*_, though the extinction potential decreases with floral attractiveness (blue cells in the left panels of Fig. 6). When the plant has a high recruitment capacity (*K*_*X*_) and the insect has a low recruitment capacity (*K*_*Y*_), not only the insect, but also the plant, becomes extinct (black cells in the left panels of Fig. 6). Furthermore, the effects of recruitment capacity vary with floral attractiveness. If floral attractiveness is relatively low (*a*_*M*_ = *a*_*F*_ = 0.2) the plant goes extinct in a large parameter space, and only the insect survives when it has a sufficiently high recruitment capacity (blue cells in Fig. 6A). When floral attractiveness is moderately low (*a*_*M*_ = *a*_*F*_ = 0.4) coexistence can be achieved in some parameter space; however, the plant becomes extinct counterintuitively when the insect has a high recruitment capacity (blue cells in Fig. 6C). This occurs because pollination success saturates even if the numbers of the insect increase, whereas an increase in female insects prevents male pollinators from being trapped by female plants (i.e. dilution effects). If floral attractiveness is higher, the plant becomes extinct even when it has a high recruitment capacity, because the insect becomes extinct owing to a low recruitment capacity (black cells in Fig. 6E and G). In the second scenario (i.e. *r*_*VM*_ = *r*_*VF*_ > 0 and *g*_*MF*_ is high), however, plants rarely become extinct because they have the capability of vegetative reproduction (right panels of Fig. 6). Meanwhile, the insects become extinct because of deceptive pollination when the plant and insect have high and low recruitment capacities, respectively, and the parameter space expands with floral attractiveness (green cells in the right panels of Fig. 6).

The effects of plant death rates are also distinct between the two scenarios (Fig. 7). In the first scenario (*r*_*VM*_ = *r*_*VF*_ = 0 and *g*_*MF*_ is low), the plant tends to become extinct when both male and female plants have high death rates (blue cells in the right panels of Fig. 7) and co-extinction occurs when female plants have a relatively low death rate (black cells in the left panels of Fig. 7). Plant extinction from high death rates is less likely to occur when floral attractiveness is high. However, the potential for co-extinction counterintuitively increases under extremely low death rates of female plants as a result of high floral attractiveness (black cells in Fig. 7E, G). This is because male insects are trapped by female plants, which leads to the subsequent extinction of the plant without vegetative reproduction capability. In the second scenario (*r*_*VM*_ = *r*_*VF*_ > 0 and *g*_*MF*_ is high), the plant is less likely to become extinct (compare blue cells between left and right panels of Fig. 7). Co-extinction can also occur for moderately low death rates when floral attractiveness is high (black cells in Fig. 7H). This would be caused by the same mechanism as in the first scenario. In the second scenario, however, the plant can persist through vegetative reproduction and only the insect becomes extinct because of deceptive pollination when the death rate of female plants is low (green cells in the right panels of Fig. 7). The parameter space for insect extinction expands with increasing floral attractiveness (green cells in the right panels of Fig. 7).

## DISCUSSION

Plant–pollinator relationships have contributed significantly to the Earth’s biodiversity (Eriksson and Bremer, 1992; Vamosi and Vamosi, 2010; van der Niet and Johnson, 2012), evolving into various forms over time (Fægri and van der Pijl, 1979; Dafni, 1984; Johnson, 2010). In this study we have theoretically investigated the population dynamics and disturbance responses of lethal deceptive pollination systems in *Arisaema* species, representing the most extreme case of parasitic plant–pollinator relationships. Our results demonstrated that these deceptive plants can reduce pollinator abundance and even lead to the extinction of themselves and/or their pollinators. Indeed, we often observe very low visitation frequencies of fungus gnat pollinators in *Arisaema* populations (T. K. Matsumoto, personal observations; see also Bierzychudek, 1982; Nishizawa *et al.*, 2005; Barriault *et al.*, 2009). These observations may indirectly support the prediction that fungus gnat populations could be significantly impacted by trapping by *Arisaema* species, although the quantitative evidence has not yet been established. It is therefore suggested that the demographic vulnerability of *Arisaema* species would differ qualitatively from that of general mutualistic pollination systems, where plants benefit pollinators. This underscores the necessity for special considerations in the conservation management of endangered *Arisaema* species.

### Two major scenarios of biological parameters in lethal deceptive pollination

Our model predicted that the population dynamics of the lethal deceptive pollination system would be qualitatively distinct depending on the rates of vegetative reproduction and transition from male to female (Figs 4–7). These parameters can critically affect the exploitation of pollinators by deceptive plants, via increasing female plants. Through parameter calibrations based on field observational data (Fig. 3), we identified the two main scenarios for *Arisaema* species: (1) the rates of vegetative reproduction and transition from male to female are both low (first scenario), which was found for *A. angustatum*, *A. peninsulae* and *A. pseudoangustatum* var. *pseudoangustatum*; and (2) the two parameters are both relatively high (second scenario), as observed for *A. ovale*. Our model predicted that the plant in the first scenario would fail to exist unless the insect can provide sufficient pollination opportunities because it lacks vegetative reproduction capability (Figs 4–7). In this scenario, the system exhibits alternative stable states in which the plant becomes extinct or coexists with the insect, depending on the initial conditions (white cells below the solid line in Fig. 4). This implies that such plants may have difficulty colonizing new habitats or recovering from severe disturbances.

The level of floral attractiveness may differ between the two scenarios because the reliance on pollinators differs depending on the capability of vegetative reproduction. In the first scenario (i.e. the rates of both vegetative reproduction and transition from male to female are low), coexistence of the plant and insect is easier at high floral attractiveness, whereas in the second scenario (i.e. both rates are high), plant–insect coexistence is likely to be possible only when floral attractiveness is relatively low (Figs 4 and 5). These predictions imply that *A. ovale* might have lower floral attractiveness than the other three species studied here because of its low need for pollination due to high ability of vegetative reproduction (Fig. 3C; Murata, 1993; Murata *et al.*, 2018; Matsumoto *et al.*, 2020). This prediction does not contradict the intra- or interspecific gradients of floral attractiveness in other plant taxa with non-lethal deceptive pollination systems (Gaskett *et al.*, 2008; Vereecken *et al.*, 2012; de Jager and Ellis, 2014). However, it is still unclear for any *Arisaema* species to what extent the floral odour is similar to sex pheromones of female pollinators (Matsumoto *et al.*, 2021, 2023*b*; Suetsugu *et al.*, 2021). To address this question, chemical analyses of the floral odours of male and female plants and sex pheromones of female pollinators are indispensable in the future.

### Disturbance responses of lethal deceptive pollination systems and implications for conservation management

Of the 180 *Arisaema* species described worldwide, 48 species with extremely small genetic distances (section *Pistillata*) are distributed in the Japanese Archipelago (Murata *et al.*, 2018), suggesting rapid diversification of this dioecious genus (Murata and Kawahara, 1995; Ohi-Toma *et al.*, 2016). This diversity is fascinating for studying pollinator-mediated adaptive radiation (Okuyama and Kakishima, 2022). Nevertheless, many *Arisaema* species are currently endangered, and one-third of the Japanese *Arisaema* species are included on the Japanese Red List (Murata *et al.*, 2018). Forest habitat reduction, increasing deer abundance, and theft for horticultural cultivation have been discussed as the three main disturbance types in *Arisaema* species in Japan (Oshima *et al.*, 1997; Murata *et al.*, 2018; Matsumoto *et al.*, 2020). Motivated by this concern, we analysed the model to investigate how different disturbance types can differentially impact lethal deceptive pollination systems from a demographic perspective. One important prediction was that the plant would easily become extinct when the rate of vegetative reproduction is low (i.e. the first scenario; Figs 4 and 5). It was also predicted that the extinction risk of the plant without the ability of vegetative reproduction would substantially increase under these disturbances (Figs 6 and 7). These predictions can partly explain why many Japanese *Arisaema* species are currently endangered, considering that most of them (> 80 %) cannot consistently produce cormlets (Murata *et al.*, 2018). Hereafter, we will focus on the first scenario, in which plants are more likely to become extinct due to the limited capability for vegetative reproduction, to discuss the predicted vulnerability of a lethal deceptive pollination system in response to the three disturbance types.

First, we consider that an increase in deer abundance increases the plant’s recruitment capacity (*K*_*X*_) by removing other competitive plants (Fujii, 2010; Matsumoto *et al.*, 2020), while it decreases the insect’s recruitment capacity (*K*_*Y*_) by degrading forest understorey habitats and diminishing its brood sites, including mushrooms, decayed woods and soil litter (Binns, 1981; Irmler *et al.*, 1996; Jakovlev, 2012). Our model predicted that such parameter changes might cause the co-extinction of plants and insects (left panels of Fig. 6). The plant extinction could be a subsequent outcome of insect extinction caused by recruitment limitation and plant population increases. Previous studies have suggested that an increase in deer abundance may benefit *Arisaema* species by eliminating other competitive plants (Fujii, 2010; Matsumoto *et al.*, 2020). However, our results emphasize that increasing deer abundance may have a negative impact on *Arisaema* species in the long run, in contrast to previous reports, given the impact on pollinator populations. To test the prediction, it would be intriguing to compare the relative abundance of *Arisaema* plants and fungus gnat pollinators between regions with and without sika deer across the Japanese Archipelago. Further, *Arisaema* species may lower the threshold body mass for sex change when deer abundance increases (Heckel and Kalisz, 2017). Such life-history modifications may further accelerate the collapse of the deceptive pollination system due to increasing female plants, as co-extinction is likely to occur when the rate of transition from male to female is higher (Fig. 4). Therefore, conservation management of *Arisaema* species should be designed in conjunction with the demography of their pollinators.

Second, we consider that theft for horticultural cultivation would increase plant mortality, especially for female plants, because collectors prefer larger and easier-to-find plants (Table S1 in Supplementary Data Information S3). Most *Arisaema* plants change sex from male to female as they grow (Kinoshita, 1986), resulting in sexual dimorphism in plant size (Fig. 3B). In natural *Arisaema* populations, plant survival does not differ much between males and females (Bierzychudek, 1982; Kinoshita, 1987). If so, our model predicts that the plant can coexist with the insect in a limited parameter space (i.e. the narrow lower-left region of white cells in the left panels of Fig. 7). Therefore, an increase in the death rate of either male or female plants may readily cause plant extinction, rescuing pollinators (blue cells in the left panels of Fig. 7). We found that the male ratio of *Arisaema* species in herbarium specimens was often significantly lower than the expected value (Table S1 in Supplementary Data Information S3). These results suggest that sampling pressure, even for academic purposes, would be greater for large females than for small males. Indeed, researchers seem to have not always distinguished between sexes during sampling because specimen records often lack sex information (T. K. Matsumoto, personal observations). If the same is true of plant poachers, theft for horticultural cultivation in recent years (Fukai, 2007; Naito *et al.*, 2015; Murata *et al.*, 2018) can increase the extinction risk of *Arisaema* species by increasing the death rate of female plants. Female plants are usually rare (Richardson and Clay, 2001; Fig. 3C), especially in harsh environments (Levine and Feller, 2004; Heckel *et al.*, 2010). Therefore, sampling a small number of female plants can drastically bias the sex ratio of *Arisaema* species, causing unexpectedly large negative effects.

Third, we consider forest habitat reduction (Oshima *et al.*, 1997), which is expected to decrease the recruitment capacity of both plants and insects (i.e. *K*_*X*_ and *K*_*Y*_, respectively). According to our predictions, this may have limited effects on the maintenance of the lethal deceptive pollination system because plant–insect coexistence remains possible even if both *K*_*X*_ and *K*_*Y*_ decrease unless floral attractiveness is too low (white cells in Fig. 6C, E, G). In reality, sensitivity to forest habitat reduction may differ between plants and insects. *Arisaema* plants can survive without sexual reproduction for up to several decades (Bierzychudek, 1982), much longer than fungus gnats, surviving at most several months (Sasakawa and Akamatsu, 1978; Kitajima and Kawashima, 2012; Kitajima *et al.*, 2012), suggesting that fungus gnat pollinators may disappear before plant populations collapse. If the insect decreases its recruitment capacity more significantly than does the plant, forest habitat reduction will lead to their co-extinction because of pollinator deficiency (black cells in the left panels of Fig. 6). These results indicate that maintaining pollinating insects is crucial for the conservation of *Arisaema* species in reduced forest habitats. Therefore, it is important to accumulate information on suitable environmental conditions for recruitment of fungus gnat pollinators for effectively preserving their brood sites. Such information is relatively limited (Okuyama *et al.*, 2018) compared with that on habitat affinity of *Arisaema* species (Muraoka *et al.*, 1997; Marui *et al.*, 2004).

*Arisaema* species may not be able to recover easily once their populations collapse following disturbance because alternative stable states can occur in which the plant becomes extinct or coexists with the insect, depending on the initial condition (white cells below the solid line in Fig. 4). The occurrence of alternative stable states suggests that precautionary actions prior to disturbances are essential for conservation management (Scheffer and Carpenter, 2003; Folke *et al.*, 2004; Nakazawa, 2014). Therefore, it is important to not only monitor temporal changes in abiotic habitat circumstances, including light, nutrients, and water availability (Muraoka *et al.*, 1997; Levine and Feller, 2004; Heckel *et al.*, 2010), but also demographic aspects, including population abundance and sex ratio of *Arisaema* plants (Bierzychudek, 1982; Kinoshita, 1987) and fungus gnats (Sutou and Ito, 2005; Kurina and Grootaert, 2016). Such demographic monitoring is critical for detecting the precursors of population collapse, i.e. early-warning signals (Scheffer *et al.*, 2009), and developing adaptive conservation management.

Overall, we recommend multifaceted practices for the conservation of *Arisaema* species. Specifically, it is primarily important to more fully understand population dynamics and ecological niches not only of *Arisaema* species but also of fungus gnats in nature because their pollination can mediate long-term disturbance responses of the lethal deceptive pollination system. In addition, landscape-scale management needs to consider not only vegetation degradation but also brood site reduction for fungus gnats under high grazing pressure because intensive deer herbivory can have a counterintuitive impact on the lethal deceptive pollination system in the long term given the demographic responses of the pollinators. The impact of theft for horticultural cultivation can be direct and critical. Although legal protection has recently been improved to inhibit a large scale of plant theft due to increasing penalties (Murata *et al.*, 2018), relevant conservation rules and the vulnerability of *Arisaema* populations to the theft of female plants should be more widely noted. To effectively implement these practices, collaborative partnerships are required between ecological researchers, land managers and governments.

### Future perspectives

We discuss several areas for future research. We are particularly concerned with the spatial aspects, that is, spatial distribution and dispersal behaviour, of plants and insects. Although our model predicted that plants and insects would become extinct when floral attractiveness is too high (Figs 4 and 5), this negative effect may be mitigated by dispersal. Most *Arisaema* species rely on birds for long seed dispersal (Suzuki and Maeda, 2014*a*, *b*; Kobayashi *et al.*, 2017; Oishi *et al.*, 2020), whereas fungus gnats have poor dispersal abilities (Jakovlev, 2012; Hu *et al.*, 2019; Ishihara and Tagami, 2020). The long dispersal ability of *Arisaema* species implies that their populations can persist through colonization from other habitats even when they may go extinct locally. An intriguing question is the conditions under which plants and insects can coexist over space, i.e. regional coexistence. To address this question, spatially structured models need to be developed in a metacommunity context (Holyoak *et al.*, 2005; Nakazawa and Huang, 2015). Given that fungus gnats are disadvantaged in both dispersal and local survival, some mechanisms including behavioural and evolutionary adaptation would be needed to achieve regional coexistence. More detailed investigations are necessary to elucidate the ecology of fungus gnats (see below).

Second, it is important to study the behavioural and evolutionary aspects of fungus gnats. It remains unclear how often male fungus gnat pollinators mate with females and whether mating experience can change the probability of future copulation attempts. In some non-lethal deceptive pollination systems, pollinating insects often avoid habitat patches that include deceptive plants (Wong and Schiestl, 2002; Wong *et al.*, 2004; Whitehead and Peakall, 2012). Furthermore, some pollinators can discriminate between deceptive plants and their copulation mates (de Jager and Ellis, 2014). Given that lethal deceptive pollination systems of *Arisaema* species are more critical for pollinator fitness than non-lethal ones, we expected that anti-plant evolution would be more likely to occur in the pollinators of *Arisaema* species (Matsumoto *et al.*, 2021; Okuyama and Kakishima, 2022). Such behavioural modifications may help delay or prevent pollinator extinction in local populations when floral attractiveness is relatively high.

Third, we emphasize that further field observations of fungus gnats are warranted because the demographic data, including population abundance, sex ratio and life-history characteristics of pollinators of *Arisaema* species remain largely unexplored. In sexually deceptive orchids, hymenopteran pollinators often bias the sex ratio towards males via asexual reproduction, thereby counteracting the exploitation of male individuals by deceptive plants (Gaskett *et al.*, 2008; Brunton-Marin *et al.*, 2021*a*, *b*, 2022). Similarly, Brunton-Marin *et al.* (2022) suggested that the increased male ratio of sciarids under low temperatures can mitigate the negative effects of deceptive pollination. Some other studies have experimentally shown that the sex ratio of fungus gnats varies with temperature (Nigro *et al.*, 2007; Shirvani Farsani *et al.*, 2013), implying that their sex ratio may shift seasonally along with the flowering phenology of *Arisaema* species. In addition to these issues, i.e. space, behaviour, evolution and phenology, many other questions remain to be addressed regarding the lethal deceptive pollination system of *Arisaema* species and the ecology of their pollinators.

Finally, it would also be intriguing to compare population vulnerability between lethal and non-lethal deceptive pollination systems. Most deceptive plants with trap flowers usually release captured insects to ensure cross-pollination (e.g. Oelschlägel *et al.*, 2009; Phillips *et al.*, 2014; Matsumoto *et al.*, 2023*a*; but see Vogel, 1965). Therefore, deceived flowers may only waste the time of pollinating insects. If this is the case, the co-extinction of plants and insects (Figs 4–7) would be less likely to occur in non-lethal deceptive pollination systems. Our preliminary formulations demonstrated that both plant pollination success (*Q*_1_) and insect mating success (*Q*_2_) exhibit similar patterns in response to floral attractiveness or plant sex ratio (Fig. S2 in Supplementary Data Information S4). However, it is still difficult to conclude that non-lethal deceptive pollination systems are more stable, because our model includes many assumptions specific to the *Arisaema*–fungus gnat system. For example, dioecy and/or sequential hermaphrodism (i.e. sex change) is limited to very few taxa or completely absent (Freeman *et al.*, 1980; Mayo *et al.*, 1997; Renner, 2014) in major angiosperm families (Apocynaceae, Araceae, Aristolochiaceae, Begoniaceae and Orchidaceae), which include many deceptive plants (Johnson and Schiestl, 2016). A highly male-biased sex ratio of deceptive plants (Fig. 3E) was considered for calibrating model parameters, while it may be found only in *Arisaema* species among deceptive plants due to the rareness of dioecy. In addition, most sexually deceptive orchids employ hymenopteran pollinators in which females can produce male progenies without successful copulation (Brunton-Martin *et al.*, 2022). These situations indicate that the present model cannot be readily applied to simulate population dynamics in non-lethal pollination systems of other plant taxa. Alternatively, we may highlight some exceptional cases in *Arisaema* species. For example, androdioecy is sporadically found across the phylogeny of this genus (Murata *et al.*, 2018). They can reversibly change sex expression from male to bisexual (Clay, 1993). In bisexual plants, each anther begins to dehisce (male phase) after the decline of stigma receptivity (female phase) during a flowering season. Because the exit hole of the spathe tube opens when the male phase starts in bisexual *A. tortuosum* plants (Vogel and Martens, 2000), androdioecious *Arisaema* species may release captured pollinators as in non-lethal deceptive plants. On the other hand, a mycetophilid pollinator species (*Leia ishitanii*) emerges from the decayed female inflorescence of *A. thunbergii* subsp. *thunbergii* (Suetsugu *et al.*, 2024), suggesting that *Arisaema* plants may provide captured pollinators with a brood site (i.e. floral reward) in this case. Descriptive research on the natural history of these systems may provide us with a comparable model of non-lethal deceptive pollination in the future.

In conclusion, the present study has highlighted the importance of incorporating natural history knowledge into population dynamics models for a better understanding of unique pollination systems and their behaviour under different types of disturbances. There has long been concern over a gap between theoretical ecology and natural history research (Caswell, 1988; Shoemaker *et al.*, 2021). We believe that bridging the gap can open a new research avenue for pollination biology and conservation management of many unique plants.

## SUPPLEMENTARY DATA

Supplementary data are available at *Annals of Botany* online and consist of the following. Information S1: method behind deriving the quantities *Q*_1_ and *Q*_2_ and the probability *P*. Information S2: sensitivity analysis by varying one parameter while fixing the three other parameters at default values. Information S3: inferring that plant poachers for horticultural cultivation would tend to collect female plants more frequently than male plants, because female plants are usually larger and easier to find. Information S4: deriving the quantities *Q*_1_ and *Q*_2_ in the case of non-lethal deceptive pollination. Figure S1: results of sensitivity analysis. For each panel, one parameter was varied, as shown in the figure. Figure S2: effects of floral attractiveness and plant sex ratio on visiting behaviour of male insects in the case of non-lethal deceptive pollination. Table S1: sex ratio of five *Arisaema* species in herbarium specimens.

mcae108_suppl_Supplementary_Figure_S1

mcae108_suppl_Supplementary_Figure_S2

mcae108_suppl_Supplementary_Material

mcae108_suppl_Supplementary_Table_S1

## References

[CIT0001] Augustine DJ , McNaughton SJ. 1998. Ungulate effects on the functional species composition of plant communities: herbivore selectivity and plant tolerance. Journal of Wildlife Management 62: 1165–1183.

[CIT0002] Baguette M , Bertrand JAM, Stevens VM, Schatz B. 2020. Why are there so many bee-orchid species? Adaptive radiation by intra-specific competition for mnesic pollinators. Biological Reviews 95: 1630–1663. 32954662 10.1111/brv.12633

[CIT0003] Barriault I , Gibernau M, Barabé D. 2009. Flowering period, thermogenesis, and pattern of visiting insects in *Arisaema triphyllum* (Araceae) in Quebec. Botany 87: 324–329.

[CIT0004] Bascompte J , Jordano P. 2014. Mutualistic networks. Princeton: Princeton University Press.

[CIT0005] Bierzychudek P. 1982. The demography of jack-in-the-pulpit, a forest perennial that changes sex. Ecological Monographs 52: 335–351.

[CIT0006] Bierzychudek P. 1984. Determinants of gender in jack-in-the-pulpit: the influence of plant size and reproductive history. Oecologia 65: 14–18. 28312103 10.1007/BF00384456

[CIT0007] Binns ES. 1981. Fungus gnats (Diptera: Mycetophilidae/Sciaridae) and the role of mycophagy in soil: a review. Revue d’Ecologie et de Biologie du Sol 18: 77–90.

[CIT0008] Bohman B , Flematti GR, Barrow RA, Pichersky E, Peakall R. 2016. Pollination by sexual deception — it takes chemistry to work. Current Opinion in Plant Biology 32: 37–46. 27368084 10.1016/j.pbi.2016.06.004

[CIT0009] Bronstein JL. 2015. Mutualism. Oxford: Oxford University Press.

[CIT0010] Brunton Martin AL , O’Hanlon JC, Gaskett AC. 2020. Orchid sexual deceit affects pollinator sperm transfer. Functional Ecology 34: 1336–1344.

[CIT0011] Brunton Martin AL , Gaskett AC, O’Hanlon JC. 2021a. Museum records indicate male bias in pollinators of sexually deceptive orchids. The Science of Nature 108: 25. 10.1007/s00114-021-01737-x 34091791

[CIT0012] Brunton-Martin AL , Gaskett AC, Kokko H. 2021b. Resilience of haplodiploids to being exploited by sexually deceptive plants. Oikos 130: 2053–2063.

[CIT0013] Brunton-Martin AL , O’Hanlon JC, Gaskett AC. 2022. Are some species ‘robust’ to exploitation? Explaining persistence in deceptive relationships. Evolutionary Ecology 36: 321–339.

[CIT0014] Caswell H. 1988. Theory and models in ecology: a different perspective. Ecological Modelling 43: 33–44.

[CIT0015] Clay K. 1993. Size-dependent gender change in green dragon (*Arisaema dracontium*; Araceae). American Journal of Botany 80: 769–777. 10449396

[CIT0016] Dafni A. 1984. Mimicry and deception in pollination. Annual Review of Ecology and Systematics 15: 259–278.

[CIT0017] de Jager ML , Ellis AG. 2014. Costs of deception and learned resistance in deceptive interactions. Proceedings of the Royal Society B 281: 20132861. 24478302 10.1098/rspb.2013.2861 PMC3924078

[CIT0018] Eriksson O , Bremer B. 1992. Pollination systems, dispersal modes, life forms, and diversification rates in angiosperm families. Evolution 46: 258–266. 28564968 10.1111/j.1558-5646.1992.tb02000.x

[CIT0019] Fægri K , van der Pijl L. 1979. The principles of pollination ecology, 3rd edn. Oxford: Pergamon Press.

[CIT0020] Folke C , Carpenter S, Walker B, et al 2004. Regime shifts, resilience, and biodiversity in ecosystem management. Annual Review of Ecology, Evolution, and Systematics 35: 557–581.

[CIT0021] Frederickson ME. 2013. Rethinking mutualism stability: cheaters and the evolution of sanctions. Quarterly Review of Biology 88: 269–295. 24552098 10.1086/673757

[CIT0022] Freeman DC , Harper KT, Charnov EL. 1980. Sex change in plants: old and new observations and new hypotheses. Oecologia 47: 222–232. 28309476 10.1007/BF00346825

[CIT0023] Fujii S. 2010. Changes in the understory flora following Sika deer browsing with special reference to flowering stage in Makura-dani, Ashiu Experimental Forest. Japanese Journal of Conservation Ecology 15: 3–15 [in Japanese with English abstract].

[CIT0024] Fukai S. 2007. A morphological study on *Arisaema sikokianum* (Araceae). Technical Bulletin of the Faculty of Agriculture, Kagawa University 59: 15–25 [in Japanese with English abstract].

[CIT0025] Fukushima K , Sakaguchi S, Inoue M, et al 2014. Deer overgrazing affects soil nitrogen dynamics and nitrate leaching in a cool-temperate forest ecosystem. Journal of the Japanese Society of Revegetation Technology 39: 360–367 [in Japanese with English abstract].

[CIT0026] Furusawa H , Miyanishi H, Kaneko S, Hino T. 2003. Movement of soil and litter on the floor of a temperate mixed forest with an impoverished understory grazed by deer (*Cervus nippon centralis* Temminck). Journal of Japanese Forest Society 85: 318–325 [in Japanese with English abstract].

[CIT0027] Gaskett AC , Winnick CG, Herberstein ME. 2008. Orchid sexual deceit provokes ejaculation. American Naturalist 171: E206–E212. 10.1086/587532 18433329

[CIT0028] Hashimoto Y , Fujiki D. 2014. List of food plants and unpalatable plants of sika deer (*Cervus nippon*) in Japan. Humans and Nature 25: 133–160 [in Japanese].

[CIT0029] Heckel CD , Kalisz S. 2017. Life history trait divergence among populations of a non-palatable species reveals strong non-trophic indirect effects of an abundant herbivore. Oikos 126: 604–613.

[CIT0030] Heckel CD , Bourg NA, McShea WJ, Kalisz S. 2010. Nonconsumptive effects of a generalist ungulate herbivore drive decline of unpalatable forest herbs. Ecology 91: 319–326. 20391995 10.1890/09-0628.1

[CIT0031] Holland JN , DeAngelis DL. 2010. A consumer–resource approach to the density-dependent population dynamics of mutualism. Ecology 91: 1286–1295. 20503862 10.1890/09-1163.1

[CIT0032] Holyoak M , Leibold MA, Holt RD. 2005. Metacommunities: spatial dynamics and ecological communities. Chicago: University of Chicago Press.

[CIT0033] Hu JR , Xie C, Shi CH, et al 2019. Effect of sex and air temperature on the flight capacity of *Bradysia odoriphaga* (Diptera: Sciaridae). Journal of Economic Entomology 112: 2161–2166. 31165857 10.1093/jee/toz152

[CIT0034] Irmler U , Heller K, Warning J. 1996. Age and tree species as factors influencing the populations of insects living in dead wood (Coleoptera, Diptera: Sciaridae, Mycetophilidae). Pedobiologia 40: 134–148.

[CIT0035] Ishihara Y , Tagami Y. 2020. Estimating the dispersal ability of *Bradysia odoriphaga* (Diptera: Sciaridae). Japanese Journal of Applied Entomology and Zoology 64: 107–113 [in Japanese with English abstract].

[CIT0036] Jakovlev J. 2012. Fungal hosts of mycetophilids (Diptera: Sciaroidea excluding Sciaridae): a review. Mycology 3: 11–23.

[CIT0037] Jersáková J , Johnson SD, Kindlmann P. 2006. Mechanisms and evolution of deceptive pollination in orchids. Biological Reviews 81: 219–235. 16677433 10.1017/S1464793105006986

[CIT0038] Johnson SD. 2010. The pollination niche and its role in the diversification and maintenance of the Southern African flora. Philosophical Transactions of the Royal Society B: Biological Sciences 365: 499–516. 10.1098/rstb.2009.0243 PMC2838267 20047876

[CIT0039] Johnson SD , Schiestl FP. 2016. Floral mimicry. Oxford: Oxford University Press.

[CIT0040] Kakishima S , Sueyoshi M, Okuyama Y. 2020. Floral visitors of critically endangered *Arisaema cucullatum* (Araceae) endemic to Kinki region of Japan. Bulletin of the National Museum of Nature and Science. Series B, Botany 46: 47–53.

[CIT0041] Ke PJ , Nakazawa T. 2018. Ontogenetic antagonism–mutualism coupling: perspectives on resilience of stage-structured communities. Oikos 127: 353–363.

[CIT0042] Kearns CA , Inouye DW, Waser NM. 1998. Endangered mutualisms: the conservation of plant-pollinator interactions. Annual Review of Ecology and Systematics 29: 83–112.

[CIT0043] Kinoshita E. 1986. Size-sex relationship and sexual dimorphism in Japanese *Arisaema* (Araceae). Ecological Research 1: 157–171.

[CIT0044] Kinoshita E. 1987. Sex change and population dynamics in *Arisaema* (Araceae) I. *Arisaema serratum* (Thunb.) Schott. Plant Species Biology 2: 15–28.

[CIT0045] Kitajima H , Kawashima Y. 2012. Effects of food supply and temperature on adult longevity and fecundity of *Neoempheria ferruginea*, a harmful mushroom fly in *Lentinula edodes* sawdust-based cultivation. Journal of the Japanese Forest Society 94: 209–213 [in Japanese with English abstract].

[CIT0046] Kitajima H , Ohya E, Kawashima Y. 2012. Effects of shiitake mycelial block conditions, day-length and temperature on the development of *Neoempheria ferruginea* (Brunetti) (Diptera: Mycetophilidae). Japanese Journal of Applied Entomology and Zoology 56: 1–7 [in Japanese with English abstract].

[CIT0047] Kobayashi T , Kitamura S, Murata J. 2017. Differentiation of fruiting phenology and seed dispersal of *Arisaema* (Araceae) in Japan: the effect of fruiting season on the rates of fruit removal by avian frugivores. Journal of Japanese Botany 92: 199–213 [in Japanese with English abstract].

[CIT0048] Kurina O , Grootaert P. 2016. Fungus gnats in the Botanical garden Jean Massart on the outskirts of Brussels: 52 new country records and a pictorial atlas of the genera (Diptera: Sciaroidea). Belgian Journal of Entomology 44: 1–34.

[CIT0049] Lande R , Engen S, Saether BE. 2003. Stochastic population dynamics in ecology and conservation. Oxford: Oxford University Press.

[CIT0050] Levine MT , Geller IC. 2004. Effects of forest age and disturbance on population persistence in the understory herb, *Arisaema triphyllum* (Araceae). Plant Ecology 172: 73–82.

[CIT0051] Lovett Doust J , Cavers PB. 1982. Sex and gender dynamics in jack-in-the-pulpit, *Arisaema triphyllum* (Araceae). Ecology 63: 797–808.

[CIT0052] Marui H , Yamazaki T, Umehara T, Kurosaki N, Kobayashi T. 2004. Conservation of threatened plant species *Arisaema minus* (Serizawa) J. Murata by transplantation I. Habitat and requirement for transplantation. Japanese Journal of Conservation Ecology 9: 173–182 [in Japanese with English abstract].

[CIT0053] Matsumoto TK. 2021. Application of multivariate morphometrics to delimit three Japanese species of *Arisaema* sect. *Pistillata* (Araceae). Nordic Journal of Botany 39: e03075.

[CIT0054] Matsumoto TK , Hirobe M, Akaji Y, Miyazaki Y. 2020. Population structures and spatial patterns of two unpalatable *Arisaema* species (Araceae) with and without clonal reproduction in a riparian forest intensively grazed by sika deer. Journal of Forestry Research 31: 155–162.

[CIT0055] Matsumoto TK , Hirobe M, Sueyoshi M, Miyazaki Y. 2021. Selective pollination by fungus gnats potentially functions as an alternative reproductive isolation among five *Arisaema* species. Annals of Botany 127: 633–644. 33263745 10.1093/aob/mcaa204 PMC8052922

[CIT0056] Matsumoto TK , Onoue M, Miyake T, et al 2023a. Gall midge pollination and ant-mediated fruit dispersal of *Pinellia tripartita* (Araceae). Plant Ecology 224: 59–72.

[CIT0057] Matsumoto TK , Sueyoshi M, Sakata S, Miyazaki Y, Hirobe M. 2023b. Two closely related species of the *Arisaema ovale* group (Araceae) selectively attract male fungus gnats of different *Anatella* species (Diptera: Mycetophilidae). Plant Systematics and Evolution 309: 4.

[CIT0058] Matsumoto TK , Fujisato R, Sugiyama M, Miyazaki Y, Murata J. 2024. A malformation of sex-changing plant *Arisaema serratum* (Araceae) produces both male and female inflorescences. Botany Letters 171: 102–108.

[CIT0059] Mayo SJ , Bogner J, Boyce PC. 1997. The genera of Araceae. London: Royal Botanic Gardens, Kew.

[CIT0060] Méndez M. 2001. Sexual mass allocation in species with inflorescences as pollination units: a comparison between *Arum italicum* and *Arisaema* (Araceae). American Journal of Botany 88: 1781–1785. 21669610

[CIT0061] Morris WF , Vázquez DP, Chacoff NP. 2010. Benefit and cost curves for typical pollination mutualisms. Ecology 91: 1276–1285. 20503861 10.1890/08-2278.1

[CIT0062] Muraoka H , Tang Y, Koizumi H, Washitani I. 1997. Combined effects of light and water availability on photosynthesis and growth of *Arisaema heterophyllum* in the forest understory and an open site. Oecologia 112: 26–34. 28307371 10.1007/s004420050279

[CIT0063] Murata J. 1990. Introduction to the plants of *Arisaema* recently recognized from Japan. Aroideana 13: 34–43.

[CIT0064] Murata J. 1993. Differentiation and taxonomy of *Arisaema ovale* Nakai and allies. Bulletin of the Botanical Society of Yamanashi 6: 2–8 [in Japanese].

[CIT0065] Murata J , Kawahara T. 1995. Allozyme differentiation in *Arisaema* (Araceae) (3) *Arisaema serratum* group (sect. *Pedatisecta*). Journal of Phytogeography and Taxonomy 42: 99–109.

[CIT0066] Murata J , Ohno J, Kobayashi T, Ohi-Toma T. 2018. *The genus* Arisaema *in Japan.* Tokyo: Hokuryukan [in Japanese].

[CIT0067] Naito A , Yuba T, Mizuno Y, Takasu H, Fujii S. 2015. New locality of *Arisaema cucullatum* M. Hotta in Kinki District. Bunrui 15: 195–197 [in Japanese].

[CIT0068] Nakazawa T. 2014. A dynamic resilience perspective toward integrated ecosystem management: biodiversity, landscape, and climate. In: Okuda N. ed. Biodiversity in aquatic systems and environments: Lake Biwa. Tokyo: Springer Japan, 69–91.

[CIT0069] Nakazawa T , Huang CG. 2015. Two-species metacommunity dynamics mediated by habitat preference. Oikos 125: 1334–1341.

[CIT0070] Nakazawa T , Kishi S. 2023. Pollinator sex matters in competition and coexistence of co-flowering plants. Scientific Reports 13: 4497. 36934149 10.1038/s41598-023-31671-z PMC10024751

[CIT0071] Neuschulz EL , Mueller T, Schleuning M, Böhning-Gaese K. 2016. Pollination and seed dispersal are the most threatened processes of plant regeneration. Scientific Reports 6: 29839. 27435026 10.1038/srep29839 PMC4951728

[CIT0072] Nigro RG , Campos MCC, Perondini ALP. 2007. Temperature and the progeny sex-ratio in *Sciara ocellaris* (Diptera, Sciaridae). Genetics and Molecular Biology 30: 152–158.

[CIT0073] Nishizawa T , Watano Y, Kinoshita E, Kawahara T, Ueda K. 2005. Pollen movement in a natural population of *Arisaema serratum* (Araceae), a plant with a pitfall-trap flower pollination system. American Journal of Botany 92: 1114–1123. 21646133 10.3732/ajb.92.7.1114

[CIT0074] Oelschlägel B , Gorb S, Wanke S, Neinhuis C. 2009. Structure and biomechanics of trapping flower trichomes and their role in the pollination biology of *Aristolochia* plants (Aristolochiaceae). New Phytologist 184: 988–1002. 19761495 10.1111/j.1469-8137.2009.03013.x

[CIT0115] Ohara M. 2015. *Plant Ecology*. Tokyo: Kaiyusha [in Japanese].

[CIT0075] Ohi-Toma T , Wu S, Murata H, Murata J. 2016. An updated genus-wide phylogenetic analysis of *Arisaema* (Araceae) with reference to sections. Botanical Journal of the Linnean Society 182: 100–114.

[CIT0076] Oishi R , Maeda T, Kitamura S. 2020. Effectiveness of frugivorous birds as seed dispersers for *Arisaema serratum* (Araceae) in temperate forests of Japan: fruit removal and effects on germination. Bird Research 16: A1–A14 [in Japanese with English abstract].

[CIT0077] Økland B. 1994. Mycetophilidae (Diptera), an insect group vulnerable to forestry practices? A comparison of clearcut, managed and semi-natural spruce forests in southern Norway. Biodiversity and Conservation 3: 68–85.

[CIT0078] Økland B. 1996. Unlogged forests: important sites for preserving the diversity of mycetophilids (Diptera: Sciaroidea). Biological Conservation 76: 297–310.

[CIT0079] Økland B , Götmark F, Nordén B, Franc N, Kurina O, Polevoi A. 2005. Regional diversity of mycetophilids (Diptera: Sciaroidea) in Scandinavian oak-dominated forests. Biological Conservation 121: 9–20.

[CIT0080] Okuyama Y , Kakishima S. 2022. Possible adaptive and non-adaptive radiation in three plant genera in the Japanese archipelago. Population Ecology 64: 130–135.

[CIT0081] Okuyama Y , Okamoto T, Kjærandsen J, Kato M. 2018. Bryophytes facilitate outcrossing of *Mitella* by functioning as larval food for pollinating fungus gnats. Ecology 99: 1890–1893. 29889299 10.1002/ecy.2364

[CIT0082] Oshima K , Tang Y, Washitani I. 1997. Spatial and seasonal patterns of microsite light availability in a remnant fragment of deciduous riparian forest and their implication in the conservation of *Arisaema heterophyllum*, a threatened plant species. Journal of Plant Research 110: 321–327.

[CIT0083] Pellmyr O , Huth CJ. 1994. Evolutionary stability of mutualism between yuccas and yucca moths. Nature 372: 257–260.

[CIT0084] Phillips RD , Scaccabarozzi D, Retter BA, et al 2014. Caught in the act: pollination of sexually deceptive trap-flowers by fungus gnats in *Pterostylis* (Orchidaceae). Annals of Botany 113: 629–641. 24366109 10.1093/aob/mct295 PMC3936588

[CIT0085] R Development Core Team. 2018. R: a language and environment for statistical computing. Vienna: R Foundation for Statistical Computing.

[CIT0086] Renner SS. 2014. The relative and absolute frequencies of angiosperm sexual systems: dieocy, monoecy, gynodioecy, and an updated online database. American Journal of Botany 101: 1588–1596. 25326608 10.3732/ajb.1400196

[CIT0087] Richardson CR , Clay K. 2001. Sex-ratio variation among *Arisaema* species with different patterns of gender diphasy. Plant Species Biology 16: 139–149.

[CIT0088] Ripley B , Venables B, Bates DM, Hornik K, Gebhardt A, Dirth D. 2022. *Package ‘MASS’.* https://cran.r-project.org/web/packages/MASS/index.html. (8 December 2022).

[CIT0089] Rooney TP , Waller DM. 2003. Direct and indirect effects of white-tailed deer in forest ecosystems. Forest Ecology and Management 181: 165–176.

[CIT0090] Sasakawa M , Akamatsu M. 1978. A new greenhouse pest, *Bradysia agrestis*, injurious to potted lily and cucumber. Scientific Reports of Kyoto Prefectural University, Agriculture 30: 26–30 [in Japanese with English abstract].

[CIT0091] Scheffer M , Carpenter SR. 2003. Catastrophic regime shifts in ecosystems: linking theory to observation. Trends in Ecology and Evolution 18: 648–656.

[CIT0092] Scheffer M , Bascompte J, Brock WA, et al 2009. Early-warning signals for critical transitions. Nature 461: 53–59. 19727193 10.1038/nature08227

[CIT0093] Schreiber S , Rudolf VHW. 2008. Crossing habitat boundaries: coupling dynamics of ecosystems through complex life cycles. Ecology Letters 11: 576–587. 18371091 10.1111/j.1461-0248.2008.01171.x

[CIT0094] Shirvani Farsani N , Zamani AA, Abbasi S, Kheradmand K. 2013. Effect of temperature and button mushroom varieties on life history of *Lycoriella auripila* (Diptera: Sciaridae). Journal of Economic Entomology 106: 115–123. 23448022 10.1603/ec12241

[CIT0095] Shoemaker LG , Walter JA, Gherardi LA, DeSiervo MH, Wisnoski NI. 2021. Writing mathematical ecology: a guide for authors and readers. Ecosphere 12: e03701.

[CIT0096] Suetsugu K. 2022. *Arisaema*: Pollination by lethal attraction. Plants, People, Planet, 4: 196–200.

[CIT0097] Suetsugu K , Sato R, Kakishima S, Okuyama Y, Sueyoshi M. 2021. The sterile appendix of two sympatric *Arisaema* species lures each specific pollinator into deadly trap flowers. Ecology 102: e03242. 33190280 10.1002/ecy.3242

[CIT0098] Suetsugu K , Nishigaki H, Kakishima S, Sueyoshi M, Sugiura S. 2024. Back from the dead: a fungus gnat pollinator turns *Arisaema* lethal trap into nursery. Plants, People, Planet, 6: 536–543.

[CIT0099] Sutou M , Ito MT. 2005. Emergence of Sciaridae and the other Diptera from soil in a forest of Yokohama, Japan. Edaphologia 77: 11–14 [in Japanese with English abstract].

[CIT0100] Suzuki T , Maeda N. 2014a. Frugivores of poisonous herbaceous plants *Arisaema* spp. (Araceae) in the southern Kanto district, central Japan. Journal of the Yamashina Institute for Ornithology 45: 77–91.

[CIT0101] Suzuki T , Maeda N. 2014b. Frugivores foraging on a Japanese species of the jack-in-the-pulpit, *Arisaema angustatum* (Araceae), with reference to the general framework of the links between *Arisaema* and its major frugivore groups in Japan. Biogeography 16: 79–85.

[CIT0102] Takasu H. 1988. Life history studies on *Arisaema* (Araceae) I. Growth and reproductive biology of *Arisaema* urashima Hara. Plant Species Biology 2: 29–56.

[CIT0103] Toft RJ , Harris RJ, Williams PA. 2001. Impacts of the weed *Tradescantia fluminensis* on insect communities in fragmented forests in New Zealand. Biological Conservation 102: 31–46.

[CIT0104] Valdovinos FS , Moisset de Espanés P, Flores JD, Ramos-Jiliberto R. 2013. Adaptive foraging allows the maintenance of biodiversity of pollination networks. Oikos 122: 907–917.

[CIT0105] Vamosi JC , Vamosi SM. 2010. Key innovations within a geographical context in flowering plants: towards resolving Darwin’s abominable mystery. Ecology Letters 13: 1270–1279. 20846343 10.1111/j.1461-0248.2010.01521.x

[CIT0106] Vanbergen AJ , the Insect Pollinators Initiative. 2013. Threats to an ecosystem service: pressures on pollinators. Frontiers in Ecology and the Environment 11: 251–259.

[CIT0107] van der Niet T , Johnson SD. 2012. Phylogenetic evidence for pollinator-driven diversification of angiosperms. Trends in Ecology and Evolution 27: 353–361. 22445687 10.1016/j.tree.2012.02.002

[CIT0108] Vázquez V , Barradas I. 2017. Deceptive pollination and insects’ learning: a delicate balance. Journal of Biological Dynamics 11: 299–322. 28625106 10.1080/17513758.2017.1337246

[CIT0109] Vereecken NJ , Wilson CA, Hötling S, Schulz S, Banketov SA, Mardulyn P. 2012. Pre-adaptations and the evolution of pollination by sexual deception: Cope’s rule of specialization revisited. *Proceedings of the Royal Society B* 279: 4786–4794. 10.1098/rspb.2012.1804 PMC3497092 23055065

[CIT0110] Vogel S. 1965. Kesselfallen-Blumen. Umschau für Wissenschaft und Technik 65: 12–17.

[CIT0111] Vogel S , Martens J. 2000. A survey of the function of the lethal kettle traps of *Arisaema* (Araceae), with records of pollinating fungus gnats from Nepal. Botanical Journal of the Linnean Society 133: 61–100.

[CIT0112] Whitehead MR , Peakall R. 2012. Short-term but not long-term patch avoidance in an orchid-pollinating solitary wasp. Behavioral Ecology 24: 162–168.

[CIT0113] Wong BBM , Schiestl FP. 2002. How an orchid harms its pollinator. Proceedings of the Royal Society B 269: 1529–1532. 12184821 10.1098/rspb.2002.2052 PMC1691071

[CIT0114] Wong BBM , Salzmann C, Schiestl FP. 2004. Pollinator attractiveness increases with distance from flowering orchids. Proceedings of the Royal Society B 271: S212–S214. 15252987 10.1098/rsbl.2003.0149 PMC1810010

