# Supplementary material for: When is lethal deceptive pollination maintained? A population dynamics approach
Source: Ann Bot. 2024 Aug 2;134(4):665–82. doi: 10.1093/aob/mcae108 (PMC11523630; doi:10.1093/aob/mcae108)
Supplement: mcae108_suppl_Supplementary_Figure_S1 [file mcae108_suppl_supplementary_figure_s1.docx]

**Supplementary Information 2**

Here, we performed a sensitivity analysis by varying one parameter while fixing the other parameters at default values. We did not manipulate *K_X_*, *K_Y_*, *d_M_*, and *d_F_* because these parameters were manipulated in Figures 6 and 7. We found that the results in figure 4 are generally robust to changes in the parameter values (Fig. S1). In addition, the sensitivity analysis provided important insights into how plant–insect population dynamics vary with parameters. For example, their coexistence becomes less likely when *A_Y_* increases or when *N* decreases (Fig. S1). This is because the insect population readily goes extinct when mating opportunities are limited. Coexistence becomes less likely when *q* increases (Fig. S1) because female plants more significantly increase the mortality of male insects by trapping. By contrast, coexistence becomes more likely when *g_JM_* decreases (Fig. S1) because the insect can avoid deceptive pollination when juvenile plants grow slowly or when male plants more frequently regresses to the juvenile stage. Meanwhile, the plant tends to be highly male-biased when it coexists with the insect (Fig. S1). The male-biased sex ratio in the coexistence region is consistent with our field observational data (Fig. 3d).

**Figure S1**

Results of sensitivity analysis. For each panel, one parameter was varied as shown in the figure. The left panels show species composition, with notations being the same as those in Figure 4. Right panels show plant male ratio, with red and blue colours indicating male-biased and female-biased sex ratios, respectively. In each panel, the x and y axes represent the transition rate from male to female (*g_MF_*) and vegetative reproduction rate (*r_V,M_* = *r_V,F_*), respectively.
