# Supplementary material for: When is lethal deceptive pollination maintained? A population dynamics approach
Source: Ann Bot. 2024 Aug 2;134(4):665–82. doi: 10.1093/aob/mcae108 (PMC11523630; doi:10.1093/aob/mcae108)
Supplement: mcae108_suppl_Supplementary_Figure_S2 [file mcae108_suppl_supplementary_figure_s2.docx]

**Supplementary Information 4**

Here, we derive the quantities *Q*_1_ and *Q*_2_ in the case of non-lethal deceptive pollination. Because male insects can fulfil the life expectancy without being trapped by female plants, we do not need to calculate the expected probability that male insects visit female plants (i.e. *P* = 0). We assume that one male insect visits male plants, female plants, and female insects with the probabilities *p_M_*, *p_F_*, and *p_Y_*, respectively, where *p_M_* + *p_F_* + *p_Y_* = 1 (see the main text for the definition of each probability). The total number of visits is *N*, during which he may visit female plants more than once.

First, we derive *Q*_1_, which determines the pollination success of the plant in relation to the number of male plants that one male insect visits during *N* visits. We assume that pollination success increases with visits to male plants, whereas pollen obtained at male plants are lost after pollinating a female plant and male insects need to visit male plants again so that subsequent female plants can be pollinated. With this assumption, we are now interested in the expected number of visits to male plants between two visits to female plants. Because a male insect visits female plants with the probability *p_F_*, the expected number of visits to male plants or female insects before visiting a female plant (the expected waiting time) is *p_F_*^-1^ -1 where 1 is subtracted because the female plant is visited in the *p_F_*^-1^th place. Consider that each sequence of visits to male plants or female insects, ending with a female plant, represents one “block”. In each block, a male insect visits a male plant with the probability $\frac{p_{M}}{p_{M}+p_{Y}}$ and there is expected to be in total *N*×*p_F_* blocks among *N* visits. Note also that at least one female plant should be visited in the above situation, which occurs with the probability ${1-\left( {1-p}_{F} \right)}^{N}$. Overall, the expected number of visits to male plants before he visits female plants among *N* visits is calculated as (the probability that a male insect visits at least one female plant) × (the expected number of blocks among *N* visits) × (the expected number of visits to male plants per block). That is, $Q_{1}={1-\left( {1-p}_{F} \right)}^{N}\times Np_{F}\times\left( p_{F}^{-1}-1 \right)\times\frac{p_{M}}{p_{M}+p_{Y}}=Np_{M}\left[ {1-\left( {1-p}_{F} \right)}^{N} \right]$. Next, we derive *Q*_2_, which determines the mating success of the insect in relation to the expected number of female insects that one male insect visits during *N* visits. This is simply calculated as *Q*_2_ = *N*×*p_Y_*.

We observed that *Q*_1_ monotonically increases and *Q*_2_ monotonically decreases with the floral attractiveness (Figs. S2a and S2b). These patterns indicate that floral attractiveness improves plant pollination success (Fig. S2a), thereby suppressing insect mating success (Fig. S2b). These results are qualitatively similar to those in lethal deceptive pollination, although both *Q*_1_ and *Q*_2_ seem to be higher (Figs. 2a and 2b) because female plants do not trap male insects. We also found that *Q*_1_ varies unimodally, while *Q*_2_ does not vary with the plant male sex ratio (Figs. S2c and S2d). These patterns indicate that plant pollination success is maximised at a male-biased sex ratio, as in lethal-deceptive pollination (Fig. 2d). Meanwhile, male dominance in the plant population does not affect the mating success of the insect, as long as the total population size is fixed, because female plants do not affect male insect abundance.

**Figure S2**

Effects of floral attractiveness and plant sex ratio on visiting behaviour of male insects in the case of non-lethal deceptive pollination. The upper panels show the effects of the sex-generic degree of floral attractiveness (*a_M_* = *a_F_*) on (a) the expected number of male plants one male insect visits before he visits at a female plant (*Q*_1_) and (b) the expected number of female insects one male insect visits during life expectancy (*Q*_2_), respectively. For presentation purposes, the male ratio of the plant and insect is fixed at 0.8 and 0.5, respectively. The bottom panels show the effects of male ratio of the plant on (c) *Q*_1_ and (d) *Q*_2_, respectively. For presentation purposes, the sex-generic floral attractiveness is fixed at *a_M_* = *a_M_* = 0.4 and the abundance of female insects is fixed at *Y_F_* = *K_Y_*/2. The dashed, solid, and thick lines indicate *N* = 5, 10, and 20, respectively.
