# Supplementary material for: When is lethal deceptive pollination maintained? A population dynamics approach
Source: Ann Bot. 2024 Aug 2;134(4):665–82. doi: 10.1093/aob/mcae108 (PMC11523630; doi:10.1093/aob/mcae108)
Supplement: mcae108_suppl_Supplementary_Material [file mcae108_suppl_supplementary_material.docx]

**Supplementary Information 1**

Here, we explain how to derive the quantities *Q*_1_ and *Q*_2_ and the probability *P*. First, we derive *Q*_1_, which determines the pollination success of the plant in relation to the number of male plants that one male insect visits until he dies at a female plant. For deceptive pollination to occur, a male insect finally visits a female plant with probability *p_F_*. Before that, he has at most *N*-1 times of events to visit male plants or female insects. Assume that he has in total *j* times of visit events and visits male plants *k* times; that is, he visits female insects *j-k* times. The expected number of male plants an male insect visits until he dies at a female plant is calculated as $\sum_{k=0}^{j} {k(}_{j}C_{k})\left( p_{M} \right)^{k}\left( p_{Y} \right)^{j-k}$ where *k* is the number of visiting male plants and $(_{j}C_{k})\left( p_{M} \right)^{k}\left( p_{Y} \right)^{j-k}$ is the probability of visiting male plants *k* times among *j* visiting events. By taking the summation for *j* = 0, 1, 2, … *N*-1, we obtain $Q_{1}=p_{F}\sum_{j=0}^{N-1} \sum_{k=0}^{j} {k(}_{j}C_{k})\left( p_{M} \right)^{k}\left( p_{Y} \right)^{j-k}$.

Next, we derive *Q*_2_, which determines the mating success of the insect in relation to the number of female insects that one male insect visits until he dies at a female plant. There are two cases in which he can mate with female insects. In one case, he is finally trapped by a female plant after mating and cannot live for his maximum lifetime. In the other case, he reaches his maximum lifetime without being trapped by a female plant. In the first case, he has, at most, *N*-1 events visiting male plants or female insects. The expected number of female insects an male insect visits until he dies at a female plant is formulated as $\sum_{j=0}^{N-1} \sum_{k=0}^{j} {k(}_{j}C_{k})\left( p_{Y} \right)^{k}\left( p_{M} \right)^{j-k}$ as with *Q*_1_. Thereafter, he dies at the female plant with probability *p_F_*. In the second case, a male insect has *N* times of events to visit male plants or female insects. The expected number of visiting female insects during his lifetime is calculated as $\sum_{k=0}^{N} {k(}_{N}C_{k})\left( p_{Y} \right)^{k}\left( p_{M} \right)^{N-k}$ wherein *k* is the number of visiting female insects and *_N_C_k_(p_Y_)^k^(p_M_)^N-k^* is the probability of visiting female insects *k* times among *N* visiting events. By summing these two terms, we obtain $Q_{2}=p_{F}\sum_{j=0}^{N-1} \sum_{k=0}^{j} {k(}_{j}C_{k})\left( p_{Y} \right)^{k}\left( p_{M} \right)^{j-k}+\sum_{k=0}^{N} {k(}_{N}C_{k})\left( p_{Y} \right)^{k}\left( p_{M} \right)^{N-k}$.

Finally, we derive *P*, the expected probability that one male insect visits and dies at a female plant during his lifetime. Before a male insect finally visits a female plant with the probability *p_F_*, he has at most *N*-1 times of events to visit male plants or female insects. The expected probability that he does not visit female plants at all among *k* visiting events is calculated as $\left( {1-p}_{F} \right)^{k}$. By taking the summation for *k* = 0, 1, 2, … *N*-1 and multiplying by the probability of visiting a female plant at the end, we obtain $P=p_{F}\sum_{k=0}^{N-1} \left( p_{M}+p_{Y} \right)^{k}$.
