# Supplementary material for: When is lethal deceptive pollination maintained? A population dynamics approach
Source: Ann Bot. 2024 Aug 2;134(4):665–82. doi: 10.1093/aob/mcae108 (PMC11523630; doi:10.1093/aob/mcae108)
Supplement: mcae108_suppl_Supplementary_Table_S1 [file mcae108_suppl_supplementary_table_s1.docx]

**Supplementary Information 3**

Here, we infer that plant poachers for horticultural cultivation would tend to collect female plants more frequently than male plants, because female plants are usually larger and easier to find (Fig. 3b). Although we could not directly test this, we examine whether the sex ratio of herbarium specimens is significantly more female-biased than that of the natural populations. We surveyed the sex of dry specimens of *A. japonicum* (*n* = 57), *A. minus* (*n* = 66), *A. sikokianum* (*n* = 167), *A. tosaense* (*n* = 149), and *A. yamatense* (*n* = 131) in the herbaria of The Museum of Nature and Human Activities, Hyogo; Kurashiki Museum of Natural History; The Kochi Prefectural Makino Botanical Garden; and Tokushima Prefectural Museum. When sex records were unavailable, sex expression was assessed based on spadix morphology. Specimens were excluded from the analysis if they could not be sexed. We conducted χ^2^ tests using the ‘chisq.test’ function in R to compare the sex ratio of herbarium specimens and the mean sex ratio we obtained from natural populations of the four *Arisaema* species (male% = *c.* 75, Fig. 3d). The results showed that the sex ratio of the herbarium specimens was significantly more female-biased than expected in three of the five *Arisaema* species examined (Table S1), supporting the inference that plant poachers for horticultural cultivation may also collect female plants more frequently than male plants.

**Table S1**

The sex ratio of five *Arisaema* species in herbarium specimens.

| Sex  expression | *A. japonicum* | *A. minus* | *A. sikokianum* | *A. tosaense* | *A. yamatense* |
| --- | --- | --- | --- | --- | --- |
| Male # | 39 | 20 | 53 | 85 | 72 |
| Female # | 18 | 32 | 60 | 63 | 21 |
| Male% | 68.4 | 38.5 | 47.0 | 57.4 | 77.4 |
| *P-*value | 0.53 | **<0.001** | **<0.001** | **0.002** | 0.86 |
